# Identification of a complex genetic network underlying *Saccharomyces cerevisiae* colony morphology

**DOI:** 10.1111/j.1365-2958.2012.08192.x

**Published:** 2012-09-13

**Authors:** Karin Voordeckers, Dries De Maeyer, Elisa Zande, Marcelo D Vinces, Wim Meert, Lore Cloots, Owen Ryan, Kathleen Marchal, Kevin J Verstrepen

**Affiliations:** 1 Laboratory for Systems Biology, VIB, Bio-IncubatorGaston Geenslaan 1, B-3001, Leuven, Belgium; 2 Laboratory for Genetics and Genomics, Centre of Microbial and Plant Genetics (CMPG), KU LeuvenGaston Geenslaan 1, B-3001, Leuven, Belgium; 3 Department of Microbial and Molecular Systems, KU LeuvenKasteelpark Arenberg 20, 3001, Leuven, Belgium; 4 Banting and Best Department of Medical Research and Department of Molecular Genetics, Donnelly Centre, University of Toronto160 College St., Toronto, ON, Canada, M5S 3E1; 5 Department of Plant Biotechnology and Bioinformatics, Ghent University (VIB)Technologiepark 927, 9052, Gent, Belgium

## Abstract

When grown on solid substrates, different microorganisms often form colonies with very specific morphologies. Whereas the pioneers of microbiology often used colony morphology to discriminate between species and strains, the phenomenon has not received much attention recently. In this study, we use a genome-wide assay in the model yeast *Saccharomyces cerevisiae* to identify all genes that affect colony morphology. We show that several major signalling cascades, including the MAPK, TORC, SNF1 and RIM101 pathways play a role, indicating that morphological changes are a reaction to changing environments. Other genes that affect colony morphology are involved in protein sorting and epigenetic regulation. Interestingly, the screen reveals only few genes that are likely to play a direct role in establishing colony morphology, with one notable example being *FLO11*, a gene encoding a cell-surface adhesin that has already been implicated in colony morphology, biofilm formation, and invasive and pseudohyphal growth. Using a series of modified promoters for fine-tuning *FLO11* expression, we confirm the central role of Flo11 and show that differences in *FLO11* expression result in distinct colony morphologies. Together, our results provide a first comprehensive look at the complex genetic network that underlies the diversity in the morphologies of yeast colonies.

## Introduction

Long before genetic fingerprinting, brewers and bakers used differences in the morphologies of microbial colonies to discriminate between different strains of the common brewer's yeast *Saccharomyces cerevisiae*. Early reports from the Carlsberg research labs, first by Hansen in the 1890s, and later by Winge in the 1930s, show how differences in colony shape were used to discriminate different yeasts (Spencer and Spencer, 1997). Later, the same strategy was adopted by beer brewers, who used colony morphology to monitor the purity and identity of their yeast (Hall, 1971).

The enormous diversity in colony morphologies is both puzzling and intriguing. However, surprisingly little is known about the physiological and genetic principles that underlie colony formation and morphology. This is at least partly due to the common practice of studying planktonic cells in liquid culture rather than more heterogeneous colonies on solid substrates. Moreover, much of today's research is carried out with domesticated mutants that have lost the ability to form distinct colony morphologies (Mortimer and Johnston, 1986; Liu *et al*., 1996).

Recently, however, there is a renewed interest in the behaviour of feral yeasts on solid substrates. These studies revealed that yeast colonies are true multicellular communities that show a remarkable degree of differential gene expression and morphology that resembles to some degree cellular differentiation in higher multicellular organisms (Honigberg, 2011). Cellular differentiation into spores, for example, has been observed within specific regions of yeast colonies (Ohkuni *et al*., 1998; Piccirillo and Honigberg, 2010). Other studies have reported apoptosis, along with differential gene expression (Frohlich and Madeo, 2000; Minarikova *et al*., 2001), intercellular signalling (Palkova *et al*., 1997), changes in metabolism (Vachova *et al*., 2009a) and spatial organization (Varon and Choder, 2000; Scherz *et al*., 2001) in yeast colonies, indicating a higher level specialization and communication during growth on solid substrates. One particular gene, *FLO11*, which encodes a large cell-surface protein, has been identified as one of the key players in colony development (Granek and Magwene, 2010; Vachova *et al*., 2011). Interestingly, apart from being crucial for proper development of colony morphology, *FLO11* also confers adhesion of the colony to the substrate. Moreover, in nutrient-poor conditions expression of *FLO11* is necessary, but not sufficient, for the formation of pseudohyphae, which are chains of elongated cells at the edge of the colony (Gimeno *et al*., 1992; Lo and Dranginis, 1996). When yeast cells are grown on semi-solid substrates, *FLO11* is required for the formation of large, thin biofilm-like structures called ‘mats’ (Reynolds and Fink, 2001; Reynolds, 2006; Reynolds *et al*., 2008).

*FLO11* encodes a large mucin-like cell surface protein that shows homology to other *S. cerevisiae* adhesin genes, such as *FLO1*, *FLO5*, *FLO9* and *FLO10*. All Flo proteins share a common structure composed of three domains. A C-terminal glycosylphosphatidylinositol (GPI)-anchor domain allows temporary anchoring of the protein in the cell membrane. A central domain contains serine and threonine-rich tandem repeats (Verstrepen *et al*., 2005; Gemayel *et al*., 2010). Variation in repeat number in the central domain allows for changes in *FLO11*-mediated phenotypes (Verstrepen *et al*., 2005; Fidalgo *et al*., 2008). The N-terminal domain of Flo11, however, differs from that of the other Flo proteins. Flo1, Flo5, Flo9 and Flo10 contain a lectin-like binding pocket that selectively binds specific sugar residues present on the surface of other cells. This structure is absent in Flo11 and this difference explains why Flo11 does not confer cell–cell adhesion (Verstrepen and Klis, 2006; Van Mulders *et al*., 2009; Veelders *et al*., 2010; Goossens *et al*., 2011). Instead, the presence of the long, variable central Flo11 domain seemingly increases the hydrophobicity of the yeast cell wall and increases adhesion to abiotic surfaces and substrates. A recent study shows that Flo11 proteins can even be shed from the cells, forming a extracellular layer of a mucus-like substance that may facilitate sliding motility (Karunanithi *et al*., 2010).

The regulation of *FLO11* is remarkably complex. The long (3 kb) promoter of *FLO11* integrates inputs from several signalling pathways, including the MAPK and RAS-cAMP-PKA pathways, which tune *FLO11* expression in response to environmental changes (Lambrechts *et al*., 1996; Rupp *et al*., 1999; Bruckner and Mosch, 2011; Granek *et al*., 2011). A second regulatory layer employs non-coding RNAs, which yield a toggle-like bimodal expression (Bumgarner *et al*., 2009). Furthermore, *FLO11* is also regulated by changes in the chromatin state, which makes the expression state epigenetically heritable from mother to daughter cells (Halme *et al*., 2004; Octavio *et al*., 2009).

Though previous studies have shown the enormous complexity underlying yeast colony morphology and physiology, they were not systematic. In those studies, relatively few genes were directly linked to colony morphology, and they do not represent a comprehensive view of the genetic network underlying colony formation. In this study, we performed a genome-wide screen to identify all non-essential genes that affect colony morphology in the Sigma 1278b strain. Our results reveal an extremely complex genetic network, involving multiple signalling pathways, including MAPK and cAMP-PKA, the HOG pathway, the TORC1 pathway, and the entire RIM101 pathway. The network derived from this work reveals the importance of endocytosis, protein sorting and actin modification in determining colony morphology. It also indicates that tRNA acetylation could be important in the induction of an altered morphology. Moreover, our screen confirms *FLO11* as one of few effector genes that play a direct, functional role in establishing colony morphology. To further investigate the role of *FLO11*, we investigated the effects of *FLO11* expression on morphology. We show that *FLO11* expression is uniform within colonies, and that differences in overall *FLO11* expression levels are directly linked to differences in colony morphology. Lastly, we compare the gene expression profile of a wrinkly strain to that of a smooth *flo11*Δ mutant. The results show that disruption of colony morphology results in relatively few pronounced changes in gene expression, with a few notable exceptions, including genes involved in respiration and genes encoding cell surface proteins.

## Results

### Colony morphology is influenced by growth conditions

**Fig. 1:**
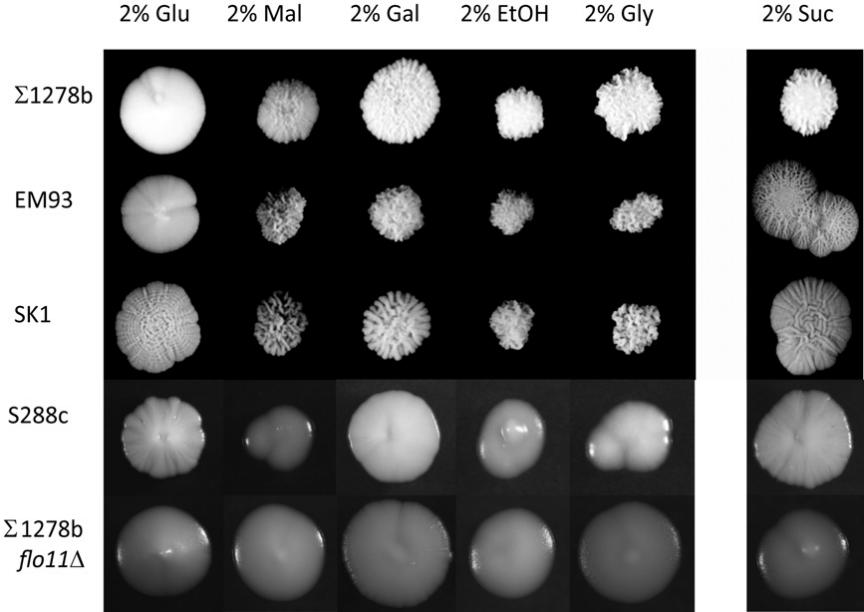
Yeast colony morphology depends on strain background and environment. Different media confer different morphologies in the same background and different strains yield different morphologies in the same media. Strains were grown in media with different carbon sources as described in *Experimental procedures*. Glu, glucose; Mal, maltose; Gal, galactose; EtOH, ethanol; Gly, glycerol; Suc, sucrose.

The most commonly used yeast research strain S288c does not show a pronounced colony morphology, presumably because it was specifically selected not to show cell–cell and cell-surface adhesion (Mortimer and Johnston, 1986). Hence, to study colony morphology, we first investigated the morphologies of various other yeast strains under several different conditions. More specifically, we grew the strains SK1, Sigma 1278b and EM93 (the feral progenitor of S288c) in different temperatures, agar concentrations, pH, carbon and nitrogen sources. The results indicate that each of these strains showed remarkably complex, strain-specific morphologies that were influenced by the environmental conditions. Notably, media with glucose repressed wrinkled morphologies, while media containing other carbon sources, such as sucrose, promoted wrinkliness (Fig. 1). Similarly, varying agar concentrations in the medium also influenced the observed colony morphologies, with low concentrations resulting in flat, biofilm-like mats. Gradual increases in agar concentrations led to a gradual reduction in the surface area of the mats and caused a gradual transition from mats to small colonies with a reduced circumference but increased height (distance from the surface of the substrate to the top of the colony) (Fig. S1).

### Colony morphology is regulated by a complex genetic network

Colony morphology is influenced by several environmental parameters, as shown in Fig. 1 and Fig. S1 for example. Some of these factors, such as the concentration of agar, may influence colony morphology by changing the physical and chemical properties of the substrate (e.g. surface tension, surface hydrophobicity etc … ). Other parameters, such as carbon source, likely act, at least in part, by changing the physiology of the yeast. Because of these multiple parameters, we hypothesized that colony morphology is likely regulated by several complex physiological processes involving many gene products.

To investigate the genetic network involved in regulating colony morphology, we examined the morphology of a set of 4156 mutants in the Sigma 1278b background, each carrying a deletion of one non-essential gene (Dowell *et al*., 2010). The morphology of each mutant was evaluated in conditions that promote the formation of complex colony morphologies (YP sucrose plates with 2% agar incubated at 30°C; see above and Fig. 1). Colonies were categorized for several criteria, including wrinkliness, size and shape (Fig. S2). Comparing the morphology of the deletion collection with the Sigma 1278b wild type, the screen identified a total of 211 gene deletions that affect morphology (52 result in smooth colonies, 159 reduce the wrinkliness) and 268 gene deletions that affect the size.

**Fig. 2:**
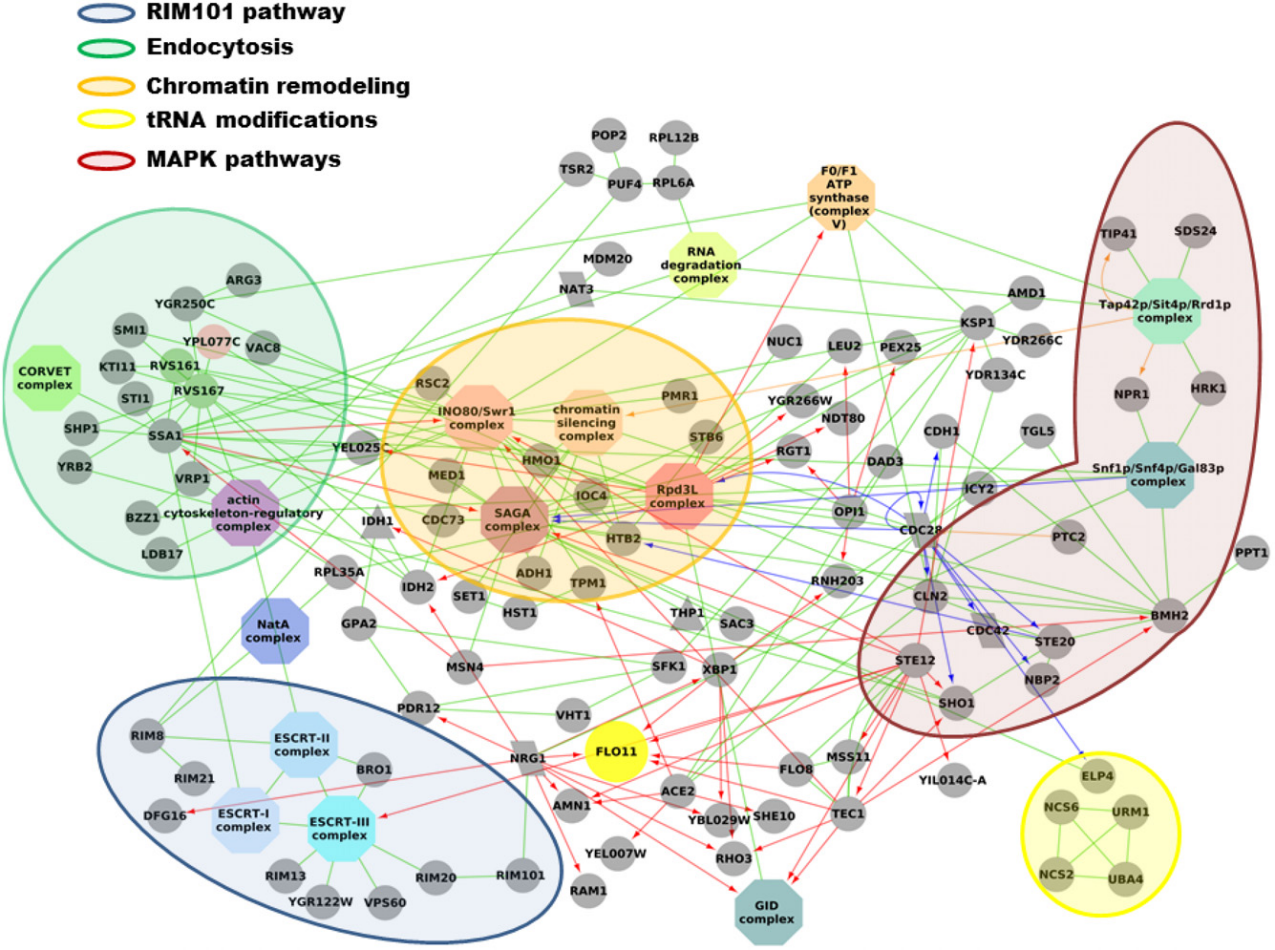
Physical interaction network visualization of genes involved in colony morphology. Genes with mutations resulting in altered colony morphology are mapped onto a physical interaction network as round nodes. To improve clarity, protein complexes containing more than 2 genes are visualized as single coloured octagonal nodes. Small protein complexes containing genes associated with colony morphology are complemented with their corresponding gene members which were not directly associated with altered colony morphology, these are visualized as a parallelogram if the mutation of this gene is lethal, or as a triangle if the mutation resulted in decreased fitness. *FLO11* is indicated as a large round yellow node. The edges between the nodes indicate physical interactions and specifically green edges indicate protein–protein interactions, blue phosphorylation interactions, orange de-phosphorylation interactions and red protein–DNA interactions. The direction if applicable for an interaction is indicated with an arrow. Genes with mutations resulting in altered colony morphology which are not connected to other smooth/semi-smooth genes or associated complexes are omitted from this figure.

Next, we used a physical interaction network to identify processes that regulate colony morphology. The 211 genes associated with altered colony morphology could be mapped onto our network (Table S1, all smooth and semi-smooth genes, minus putative proteins and dubious open reading frames). To visualize which processes and pathways play a role in colony morphology, we performed gene ontology (GO), protein complex and pathway enrichments (Tables S2 and S3, Figs S3 and S4, and File S1). The results of these analyses were mapped onto the physical interaction network to visualize the associated biological functions and processes. Figure 2 shows a simplified version of this analysis. An uncondensed version of this figure and an interactive version are available at http://homes.esat.kuleuven.be/~kmarchal/Supplementary_Information_DeMaeyer_2012_2013/yeastcolonymorphology/). The resulting network confirms previous findings that the MAPK and the RIM101 pathways play a role in colony morphology by regulating *FLO11* expression. However, our screen identifies many more genes, and several cellular processes that affect colony morphology, including chromatin modification complexes, endocytic proteins and tRNA modifying proteins.

Our screen shows that components of MAPK signal transduction pathways (Fig. 2, dark red shaded area, *P* < 1E-10 for filamentous growth (FG) associated MAPK pathway (Chavel *et al*., 2010) and *P* < 1E-5 for response to osmotic stress (GO:0006970)), the Snf1/Snf4/Gal83 complex and 10 other proteins play a role in the induction of colony morphology. Specifically, genes associated with the protein kinase C (PKC1), FG and high osmolarity glycerol (HOG) MAPK pathways (Gray *et al*., 1997; Posas *et al*., 1998; Vyas *et al*., 2003; Mapes and Ota, 2004; Saito and Tatebayashi, 2004; Saito, 2010), which largely overlap. In addition to these MAPK pathways, we also identify the Target Of Rapamycin (TOR) pathway as a central regulator of colony morphology. Similar to the MAPK cascades, the TOR pathway also plays a role in catabolite repression and stress response (Vinod and Venkatesh, 2008).

Interestingly, the pathway most tightly correlated to colony morphology in our screen is the *RIM101* pathway, which is thought to regulate gene expression in response to alkaline conditions (Fig. 2, blue shaded area, *P* < 1E-15 using a consensus pathway as described in Sarode *et al*., 2011), for an overview of all enrichments in genes associated with altered colony morphology see Table S3. Genes spanning the whole pathway (including *DFG16*, *RIM21*, *RIM8*, *SNF7*, *VPS20*, *VPS36*, *SNF8*, *STP22*, *BRO1*, *RIM13*, *RIM20*, *YGR122W* and *RIM101*) were associated with altered colony morphology, indicating a primary involvement of this signalling cascade in the regulation of colony morphology.

Another important set of genes identified in our screen as regulators of colony morphology are associated with epigenetic inheritance, chromatin modification and gene regulation (Fig. 2, orange shaded area, *P* < 1E-12 for genes in shaded area with GO term chromatin organization (GO:0006325) and *P* < 1E-10 for chromatin modification (GO:16568), for an overview of all enrichments in the all altered colony morphology associated genes see Table S3). First, we identified three genes of the Rpd3L complex (*ASH1*, *SDS3* and *SIN3*) as being involved in altered colony morphology, which is a chromatin modifying complex that plays a role in gene regulation through histone deacetylation (Carrozza *et al*., 2005). Second, three members of the Ino80/Swr1p complexes (*SWC7*, *IES3* and *ARP8*) were also identified as genes associated with colony morphology. The Ino80/Swr1 complex is ATP-dependent, and influences up to 20% of genes in *S. cerevisiae*, including genes involved in filamentation (Jonsson *et al*., 2004; Furukawa *et al*., 2011). Third, several members of the SAGA complex (*TAF12*, *SPT7*, *SUS1* and *ADA2*) were identified in our screen. The SAGA complex is involved in histone acetylation, stabilization of RNA Polymerase II and deubiquitination of histones (Grant *et al*., 1998; Koutelou *et al*., 2010). Lastly, our screen also identifies several other chromatin-related genes, including *SIR3*, *RSC2* (part of the RSC chromatin structure remodelling complex), *HTB2*, *IOC4* and *HMO1*.

Our screen also identified several genes that may influence colony morphology through post-transcriptional processes (Fig. 2, yellow shaded area, *P* < 1E-12 for genes in shaded area with GO terms wobble position uridine thiolation (GO:0002143), tRNA wobble uridine modification (GO:0002098) and tRNA wobble base modification (GO:0002097), for an overview of all enrichments in the altered colony morphology associated genes see Table S3). The protein products of these genes are related to protein tRNA modification and urmylation (Furukawa *et al*., 2000; Pedrioli *et al*., 2008). Strains defective in *UBA4* and *URM1* have been found to be defective in agar invasion (Goehring *et al*., 2003) and the tRNA modification has been linked to MAPK signalling (Abdullah and Cullen, 2009).

Several additional regulatory complexes were identified in our genetic screen. First, the glucose induced degradation (GID) complex through *GID8* and *VID24*. This complex plays a role in the regulation of the gluconeogenic processes through degradation of fructose 1,6-bisphosphatase (Santt *et al*., 2008). Second, the cytoplasmic ribosomal large subunit consisting of *RPL6A*, *RPL22A*, *RPL12B*, *RPL39*, *RPL34A* and *RPL35A*. Third, the ATP F1/F0 synthase complex consisting of *ATP18*, *OLI1*, *ATP8* and *ATP16*. Fourth, the acetyltransferases with the NatA complex consisting of *ARD1* and *NAT1*. Lastly, the NatC complex, consisting of *MAK10*, and the NatB complex, consisting of *MDM20* (Polevoda *et al*., 2003; Polevoda and Sherman, 2003).

Apart from a large set of genes that are involved in sensing, signalling and other regulatory processes, our screen also identified several genes involved in endocytosis (Fig. 2, green shaded area, *P* < 1E-11 for genes in shaded area with GO term membrane invagination (GO:0010324) and *P* < 1E-8 for endocytosis (GO:0006897), for an overview of all enrichments in the altered colony morphology associated genes see Table S3). *RVS161* and *RVS167* are associated with vesicle scission during endocytosis (Robertson *et al*., 2009; Youn *et al*., 2010). This complex plays a major role in membrane invagination, together with the protein complex Pan1/Sla1/End3 (the actin cytoskeleton-regulatory complex) and additional genes associated with membrane invagination, including *END3*, *VRP1*, *YRB2*, *LDB17* and *BZZ1* (Smythe and Ayscough, 2006; Toret and Drubin, 2006; Burston *et al*., 2009). Additionally, we identified that three members of the CORVET/HOPS complexes (*PEP5*, *VPS41 and VPS33*) play a role in altered colony morphology. These complexes can interconnect by dynamic subunit exchange and the HOPS complex has been found to play a role in the fusion of endosomes to vacuoles (Nakamura *et al*., 1997), while the CORVET complex plays a role in transition from endosome to lysosome (Peplowska *et al*., 2007).

Among the gene deletions that were shown to diminish colony morphology was only a small number of genes encoding enzymes or structural proteins. This short list includes *FLO11*, *TOS1* (encoding a cell wall protein of unknown function; Terashima *et al*., 2002) and *DFG16*, a probable multiple transmembrane sensor involved in haploid invasive growth (Mosch *et al*., 1999; Sarode *et al*., 2011). The lack of additional genes that encode structural proteins suggests that colony morphology only relies on a relatively small number of ‘effector’ genes that are directly involved in shaping a colony, and a larger number of regulatory genes.

To corroborate results obtained from this large-scale screen, we selected nine genes that were identified in the genome-wide screen as potential mediators of colony morphology and that represent different pathways and cellular processes involved. The selected genes are *DFG16* and *YGR122W* (components of the RIM101 pathway), *RVS161* (endocytosis), *SDS3*, *SWC7*, *RSC2* and *ARP8* (chromatin organization and remodelling), *URM1* (tRNA modification/protein urmylation) and *TOS1* (a cell wall protein of unknown function). Deletion of eight of these candidate genes phenocopied the results obtained in the genome-level screen and thus confirmed the involvement of these genes in colony morphology (Fig. S5A). For one gene, *TOS1*, independently created deletion mutants displayed variable phenotypes, with three mutants having a wrinkly and two having a semi-smooth colony morphology (the latter is the phenotype observed in screen). The reason for this is currently still unclear, but one possibility is rapid accumulation of suppressor mutations in a *tos1Δ* mutant, which could affect colony morphology.

In addition, we also investigated whether the marker gene used to create these gene deletions was linked to the observed phenotype. The disruption cassette used to delete the selected genes mentioned above consisted of a hygromycin resistance marker flanked by loxP sites, allowing excision of the marker gene by the Cre recombinase. In all nine cases, removal of the marker gene resulted in an exact phenocopy of the previously obtained deletion mutant (still containing the resistance marker), showing that the colony morphology phenotype is not linked to the marker gene used (Fig. S5B).

### *FLO11* is a major determinant of colony morphology

**Fig. 3:**
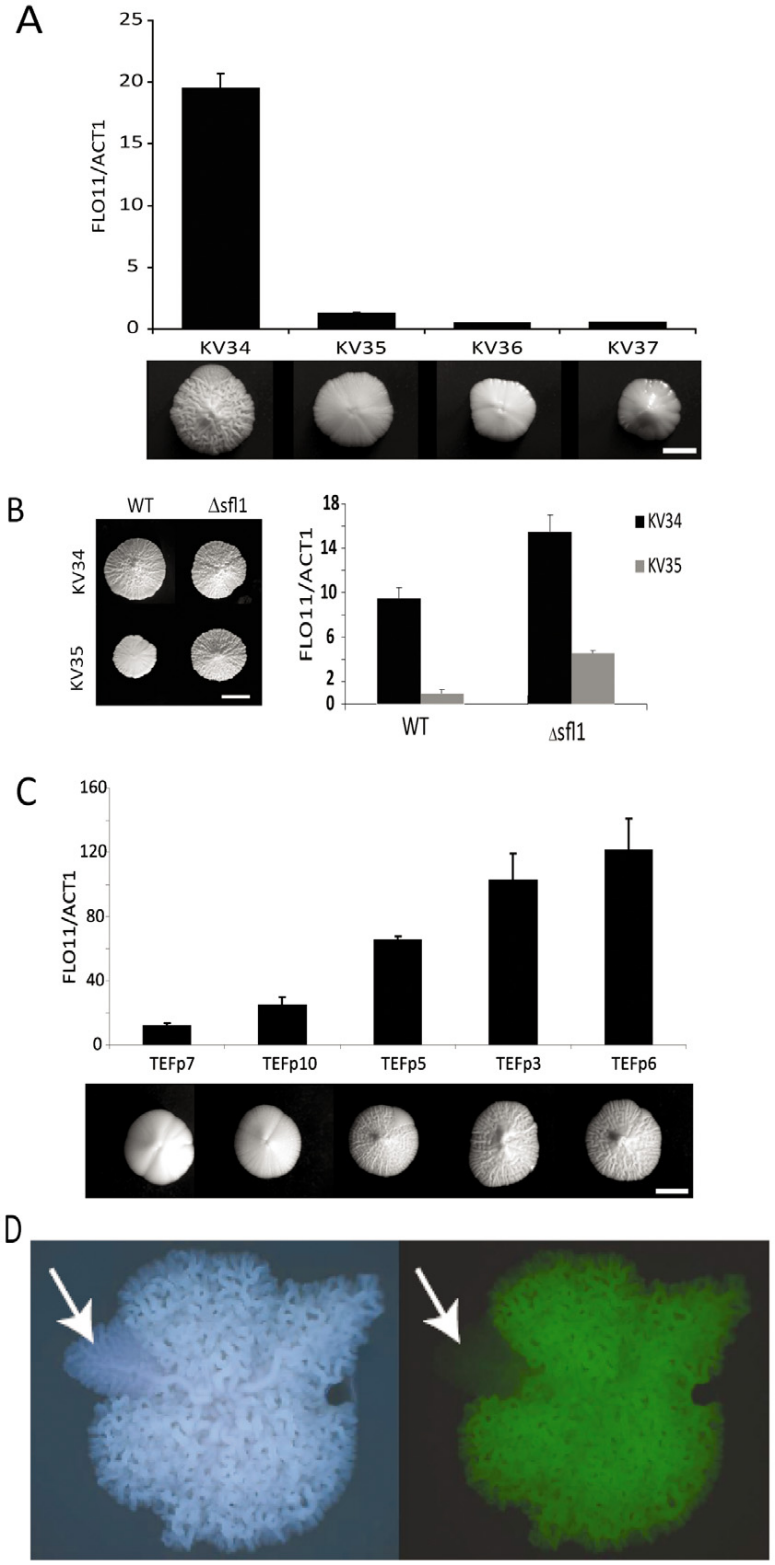
Variation of *FLO11* levels and colony morphology. A. Strains from single tetrads can also exhibit great variety in colony morphology and gene expression. Top, *FLO11* gene expression of KV34, KV35, KV36 and KV37, haploids derived from a single tetrad of EM93 diploid strain. Bottom, corresponding photos of the same strains. Both photos and gene expression levels are of colonies grown on YPS agar medium. Scale bar represents 5 mm. B. De-repressing *FLO11* expression increases wrinkliness of a smooth strain. KV34 and KV35 are sister haploid strains derived from the same tetrad of natural isolate strain EM93. KV34 is wrinkly and KV35 is smooth, and this is reflected in the levels of *FLO11* expression, with KV34 having higher levels of *FLO11*. Deletion of *SFL1*, a repressor of *FLO11* expression, raises levels of *FLO11* and makes KV35 as wrinkly as KV34. Scale bar represents 5 mm. C. Increasing *FLO11* expression correlates with increasing colony wrinkliness. Replacement of the native *FLO11* promoter by a series of constitutive promoters of increasing strength results in a series of strains with increasing wrinkliness. The TEF1prm::*FLO11* series was made in the EM93 haploid background. Scale bar represents 5 mm. D. Flo11 expression correlates with wrinkliness, but is uniform within wrinkly areas of colony. A *FLO11*–YFP construct was made that incorporated a self-cleaving viral sequence, such that simultaneous expression of Flo11 and YFP was assured without causing interference of Flo11 function. Wrinkly colonies often spawn variants or mutants with smooth morphologies, which results in smooth sectors in growing colonies (arrow in panel D). These sectors are associated with low Flo11 (low YFP) levels. However, closer inspection of wrinkly parts of colonies shows rather homogenous expression of YFP, suggesting that differential expression of *FLO11* does not account for patterned growth within a colony (bottom panel).

Since *FLO11* is one of the few downstream ‘effector’ genes that encode a protein that is directly responsible for colony morphology, and *FLO11* is downstream of a very large and complex regulatory network, we hypothesized that *FLO11* expression levels may be an important factor contributing to the diversity in colony morphologies. To investigate this possibility, we analysed the correlation between *FLO11* expression and colony morphology in a set of haploid derivatives of EM93, which is a feral diploid yeast with a pronounced colony morphology. Each haploid derivative of this heterozygous diploid feral strain shows a different colony morphology. In each of the examined haploid strains, the wrinkly phenotype correlated with the highest *FLO11* expression (one example tetrad shown in Fig. 3A). In addition, it was possible to convert a smooth haploid strain to a wrinkly strain by deleting *SFL1*, a repressor of the *FLO11* gene (Conlan and Tzamarias, 2001). Deletion of *SFL1* in this smooth strain resulted in increased *FLO11* expression and yielded wrinkly colonies that looked nearly indistinguishable from the wrinkly sister strain from the same tetrad (Fig. 3B).

Second, we constructed a series of mutants wherein we replaced the native *FLO11* promoter with a series of TEF1-derived promoters (Nevoigt *et al*., 2006), that induce a range of gene expression levels, to confirm the correlation of *FLO11* expression levels with colony morphology. The resulting strains exhibited increased colony wrinkliness that correlated with increased *FLO11* expression (Fig. 3C).

In a third experiment, we investigated *FLO11* expression in spontaneous non-wrinkly isolates derived from wrinkly progenitors. Wrinkly colonies often spawn smooth sectors within wrinkly colonies. To investigate if these non-wrinkly mutants were a consequence of *FLO11* expression, we first constructed mutants carrying a *FLO11*–YFP gene fusion. However, the strains carrying the *FLO11*–YFP fusion formed smooth colonies, indicating that tagging Flo11 with a fluorescent protein results in loss of function of Flo11. We therefore generated mutants carrying a multicistronic gene fusion of the *FLO11* gene, a self-cleaving viral peptide (picornaviral 2A peptide), and a yellow fluorescent protein (YFP) (see Experimental procedures for details). In this case, the fluorescent tag is immediately cleaved off after translation, resulting in one separate YFP molecule released in the cytoplasm for every Flo11 protein produced. The resulting strain showed normal colony morphology, indicating that the strategy to preserve Flo11 function worked. Examination of these colonies by fluorescence microscopy showed that Flo11 (as deducted from YFP levels) is present throughout the colony, except in smooth sectors, which showed virtually no fluorescence (Fig. 3D).

In a fourth experiment, the effect of deletion of the nine candidate genes mentioned above on Flo11 expression was examined, using the same cleavable *FLO11*-2A-YFP construct as discussed above (see Fig. S6). For four of these genes (*DFG16*, *YGR122W*, *URM1* and the semi-smooth *tos1Δ* mutants), deletion resulted in lower Flo11 levels, suggesting that the effect of these genes deletions may be directly caused by a reduction in Flo11 levels. Genes whose deletion did not result in reduced Flo11 levels include *RVS161* (encoding a cell raft protein involved in structural organization of the cell surface) and 4 genes (*ARP8*, *SDS3*, *SWC7* and *RSC2*) involved in chromatin modification, a process known to affect *FLO11* expression. It is possible that these genes do not affect the mean *FLO11* transcription level in the colony, but rather change the epigenetic inheritance of *FLO11* expression and/or cause differential spatial expression levels in the colony, and/or interfere with Flo11 protein processing, transport or incorporation at the cell surface. Alternatively, it is also possible that these genes do not affect *FLO11*, but rather other genes involved in colony morphology.

To investigate if, apart from *FLO11*, other genes encoding structural proteins also contribute to colony morphology, we investigated the effect of overexpression of *TOS1* and *DFG16*, two genes encoding cell surface proteins that may also play a structural role in establishing colony morphology (see above). As shown in Fig. S7, colonies overexpressing *TOS1* or *DFG16* display increased wrinkliness, which is in keep with a putative structural role for these proteins. However, further research is needed to investigate the precise contribution of Tos1 and Dfg16 to colony morphology.

### A physiological role of wrinkly colony morphology?

Why do yeast cells form such pronounced, intricate morphologies when they grow on solid substrates? Is this merely a biologically irrelevant consequence of the expression of certain cell-surface proteins such as the Flo11 adhesin? Or do the wrinkles have a biological role? To answer this question, we first tested whether there was a general fitness defect in the smooth *flo11* deletion mutants, and we tested whether smooth mutants were more or less resistant to heat and desiccation. The results of these experiments did not reveal any statistically significant difference in fitness between smooth and wrinkly colonies under the conditions tested, even though there seemed to be a trend for wrinkly colonies being more resistant (data not shown).

**Fig. 4:**
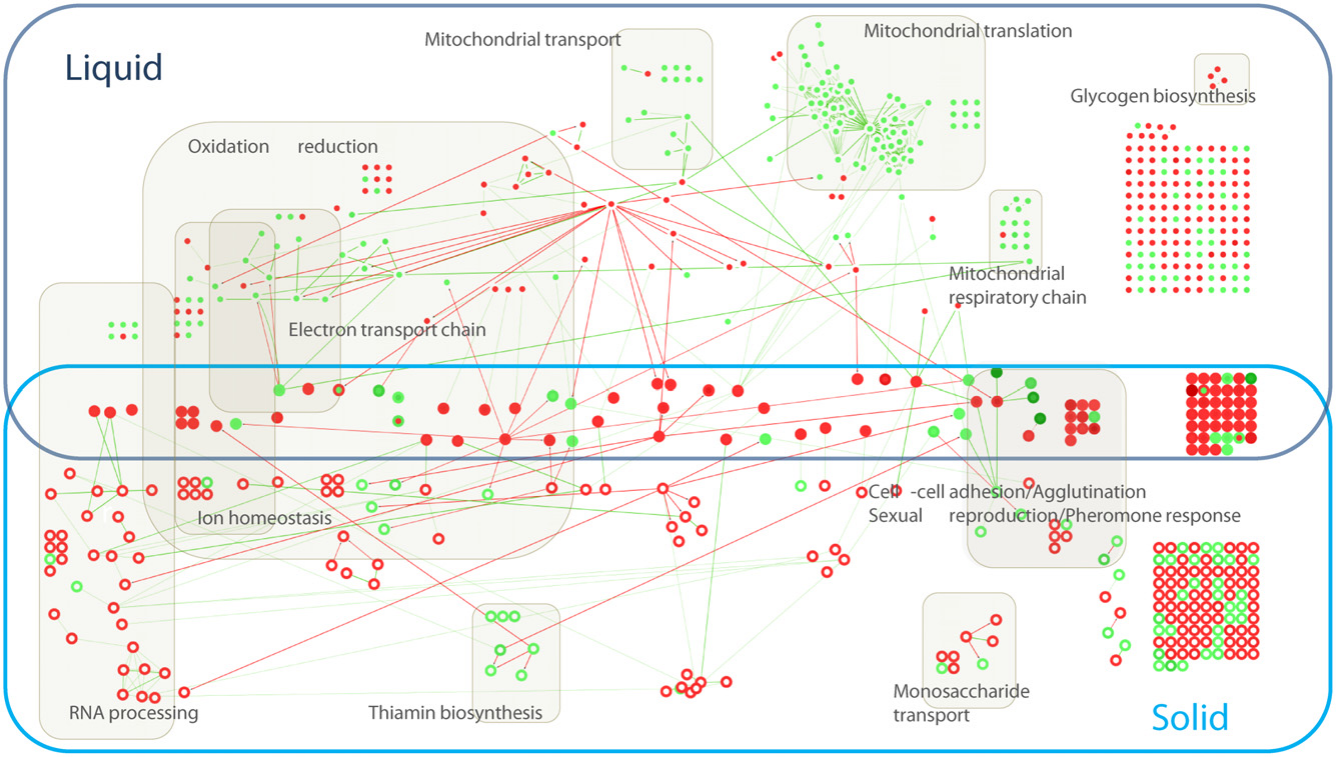
Overview of genes differentially expressed between a *flo11* deletion mutant and a wild-type strain grown on liquid and solid medium. The colour of the core of the genes indicates the differential expression of the genes in liquid, while the colour of the border indicates the differential expression on solid medium. Green indicates under-expression and red overexpression of the *flo11* mutant compared with the wild type. Overrepresented GO biological process terms were categorized and overlain onto the network as grey shaded areas. Red edges indicate protein–DNA interactions while green edges indicate protein–protein interactions.

In another approach, we hypothesized that we might be able to obtain some clues about the possible physiological relevance of wrinkly colony morphology by comparing the transcriptional response of a smooth *flo11Δ* mutant to that of a wrinkly wild-type colony. In brief, we measured the expression levels of wild-type Sigma 1278b and compared these to the expression level in a *flo11Δ* mutant by microarray. To investigate whether some of the transcriptional response to *flo11Δ* is specifically linked to growth as a colony on a solid substrate, we also performed the same comparison between the transcriptomes of planktonic wild-type and *flo11* deletion mutants grown in liquid medium. Analysis of the differentially expressed genes (Tables S4 and S5, Fig. S8 and Fig. 4, see also http://homes.esat.kuleuven.be/~kmarchal/Supplementary_Information_DeMaeyer_2012_2013/yeastcolonymorphology/) identified clusters of differentially expressed genes involved in several physiological processes. Interestingly, large clusters of genes show altered gene expression in response to *flo11* deletion in both liquid and solid medium, including genes involved in central processes like ion homeostasis, cell–cell adhesion, sexual reproduction, the electron transport chain and oxidation-reduction. Three processes are differentially regulated exclusively in solid medium: carbohydrate transport, thiamine biosynthesis and RNA processing.

## Discussion

Our comprehensive screen shows that colony morphology is regulated by a large number of genes that play central roles in the RIM101, MAPK, TOR and HOG signalling cascades. RIM101 is a pathway induced under alkaline conditions to regulate gene expression (Lamb *et al*., 2001; Hayashi *et al*., 2005; Castrejon *et al*., 2006). The MAPK and TOR pathways are involved in regulating growth, stress resistance and development (sporulation, filamentation) in response to nutrients and growth factors (for recent reviews, see (Madhani, 2000; Chen and Thorner, 2007; Hohmann, 2009; Loewith and Hall, 2011)). The HOG pathway is primarily a sensor of osmotic stress (Saito and Tatebayashi, 2004; Hohmann, 2009). Together, these results indicate that colony development is strongly influenced by environmental parameters, including pH, osmotic pressure and nutrient status. In concordance with this finding, one study has shown that hyper-osmotic stress inhibits the development of the fluffy colony morphology (Furukawa *et al*., 2009).

Apart from several major signalling pathways, colony morphology is also regulated by proteins involved in post-transcriptional regulation, tRNA modifications and endocytosis. Interestingly, though endocytosis and endosomes have not previously been linked to yeast colony formation, the homologues of some of the respective genes that were identified in our screen have been implicated in hyphae formation in *Candida albicans* (Sudbery, 2011), suggesting that it is an important process in morphogenic switching and adaptation to the environment. There is a clear link in our network between endocytosis and vacuolar sorting, most likely due to the fact that both processes rely on actin to perform their functions (Olave *et al*., 2002; Conner and Schmid, 2003; Smythe and Ayscough, 2006; Toret and Drubin, 2006; Zheng *et al*., 2009; Dion *et al*., 2010). One possibility is that endocytosis of the Flo11 cell surface adhesin may influence colony morphology (Vopalenska *et al*., 2010). However, the multitude of genes associated with endocytosis identified in this screen suggest a more complex influence of endocytosis on colony morphology.

Our results confirm that the Flo11 cell surface adhesin protein is a key player in colony development. We also note that the RIM101, cAMP/PKA and MAPK pathways that control colony morphology are also known to regulate *FLO11* expression (Pretorius and Bauer, 2002; van Dyk *et al*., 2005; Castrejon *et al*., 2006; Vinod *et al*., 2008; Granek and Magwene, 2010; Bruckner and Mosch, 2011; Granek *et al*., 2011). Similarly, it is known that chromatin modification is also involved in *FLO11* regulation (Halme *et al*., 2004; Barrales *et al*., 2008; Bumgarner *et al*., 2009; Octavio *et al*., 2009). Together, our results show that yeast colony morphology is controlled by a very large number of genes that are involved in different signalling pathways and biological processes, many of which are known to control *FLO11* regulation. Moreover, the results shown in Fig. 3 indicate that changes in *FLO11* expression levels generate differences in colony morphology. We believe that these observations at least partly explain the enormous differences in colony morphology that are observed between different *S. cerevisiae* strains. Analysis of Flo11 levels in different deletion mutants with a smooth colony morphology indicates that *FLO11* expression is necessary but not sufficient to establish a wrinkly phenotype. Our data suggest that also a correct spatial expression, proper Flo11 protein processing and/or other additional (still unknown) genes are important. Apart from Flo11, it is possible that other proteins also play a key role, and our results point at Dfg16 and Tos1 as possible candidates.

The remarkably large number of genes that are involved in regulating *FLO11* expression create an unusually large ‘mutational target size’ (i.e. the total number of DNA bases that, when mutated, result in changes in *FLO11* expression and regulation). In other words, different yeast strains are very likely to carry multiple mutations that affect *FLO11* regulation, and this may in turn affect their colony morphology. Hence, colony morphology could in fact be a rather useful proxy for genetic relatedness, indicating that the early microbiology pioneers may have had good reasons to use this criterion to distinguish between strains, isolates and mutants.

It is reassuring to see that our screen confirms some previous observations. Most notably, genes of the RIM101, cAMP and MAPK pathways have been associated with altered colony morphology (both in *S*. *cerevisiae* and *C*. *albicans*), even though these previous studies did not provide a comprehensive screen of all genes involved in colony development (Su and Mitchell, 1993; Mosch and Fink, 1997; Lamb and Mitchell, 2003; Bharucha *et al*., 2008; Jin *et al*., 2008; Granek and Magwene, 2010; Noble *et al*., 2010; Xu *et al*., 2010; Granek *et al*., 2011). It is also striking that many of the genes and pathways that control colony morphology have previously been implicated in the regulation of adhesion, mat formation, and invasive and filamentous growth (see for example Madhani, 2000; Gagiano *et al*., 2002; Verstrepen and Klis, 2006; Barrales *et al*., 2008; Reynolds *et al*., 2008; Verstrepen and Fink, 2009; Bruckner and Mosch, 2011). This suggests that all these phenomena are at least interconnected, or may even be different sides of the same physiological phenomenon.

Our study yields the first comprehensive look at the genetic network underlying yeast colony development but several central questions remain. First, though our study and previous work shows the complexity of the cellular regulation of colony morphology, it is still unclear how the various pathways translate environmental clues into specific colony morphologies. Pioneering work by Palkova and co-workers indicates that colony development depends on complex gradients in nutrients and metabolites (Palkova *et al*., 1997; 2002; Kuthan *et al*., 2003; Vachova and Palkova, 2005; Palkova and Vachova, 2006; Vachova *et al*., 2009a,b; 2011; Vopalenska *et al*., 2010). A key factor in understanding how a colony develops will require integration of our knowledge on signalling pathways with a detailed study of environmental changes in three-dimensional gradients during colony development. Given that our screen identified many genes involved in endocytosis, it is tempting to speculate that endocytosis plays a central role in colony development. Endocytosis has already been implicated to play a role in the polarized growth of cells in other fungi (Upadhyay and Shaw, 2008) which in turn affects colony morphology (Karunanithi *et al*., 2010). Clearly, further research is needed to link environmental cues to cellular changes, and to link these cellular changes to colony development.

A second series of unanswered questions revolves around the biological role of colony morphology. It is tempting to speculate that the intricate hub-and-spokes patterns may help to carry water and nutrients from the substrate through the colony, and that the wrinkled surface of a colony may help to increase the surface area for gas exchange. Whereas our transcriptome study indicated that disruption of the wrinkly pattern (by deletion of *FLO11*) does result in extensive transcriptional reprogramming, it is difficult to pinpoint specific physiological processes. Still, changes in the expression of a large number of genes involved in respiration (mitochondria, respiratory chain, ion homeostasis and oxidation/reduction; see Fig. 4) indicate that *FLO11* expression and the wrinkly colony surface may influence the balance between respiration and fermentation. Changes in expression of cell-surface genes involved in adhesion and agglutination indicate that cells adapt their cell surface in response to loss of *FLO11* expression. We hope that the genes identified in this study will propel further research into the physiological role of yeast colony formation.

## Experimental procedures

### Media

Media used in this study consisted of 1% yeast extract, 2% peptone and 2% of either glucose or sucrose (YPD or YPS). Plates of these media were made with 2% agar for standard growth conditions, and with 0.3% agar for growth on low-agar media. YPD containing Hygromycin B (Invitrogen) (200 mg l^−1^) or G418 (Formedium) (200 mg l^−1^) were used for selection of yeast transformants. Where noted glucose or sucrose were replaced with other carbon sources, such as maltose, galactose, ethanol or glycerol, to 2% final concentration.

### Genome wide screen

All deletion mutants were pinned in triplicate on 2% YPS using a Singer Rotor (Singer Instruments, UK) and grown at 30°C for 10 days before taking pictures. Pictures were assessed and all colonies were given a code based on their morphology. This allowed us to classify the genes according to the colony morphology they confer (Table S1). Gene deletions that gave an altered colony morphology (smooth, semi-smooth, extra wrinkly, small or large) were put in a direct interaction network (Fig. 2).

### Construction of the physical interaction network

Protein–protein interactions (PPI) and phosphorylation interactions were extracted from the BioGRID database (Reguly *et al*., 2006; Stark *et al*., 2006). Transcription factor–DNA interactions were obtained from Lee *et al*. (2002), Milo *et al*. (2002) and MacIsaac *et al*. (2006). Interactions are represented by edges in the network, while molecular entities (i.e. proteins and genes) are represented by nodes. Each edge (*i*,*j*) between a node *i* and a node *j* is assigned a weight *w_ij_* that reflects the probability of interaction between node *i* and *j*.

Weights for transcription factor-DNA interactions were determined as in (Yeger-Lotem *et al*., 2009). For the assignment of weights to PPI and phosphorylation interactions, a naïve Bayesian classifier, that uses the experimental technique(s) by which an interaction was measured as predictors, was implemented. To train the classifier, both a positive interaction set, consisting of literature-curated interactions measured by low-throughput techniques (Reguly *et al*., 2006), and a negative interaction set, consisting of protein pairs whose most specific co-annotation occurs in GO terms of 1000 total annotations or more (Myers *et al*., 2005), were compiled.

Additionally, phosphorylation data were added from literature curated interactions (Fiedler *et al*., 2009) and an ad-hoc probability was assigned to these interactions. Based on the probabilities assigned to edges the network was trimmed to remove interactions with low proof (Yeger-Lotem *et al*., 2009). Protein complex data were added to the network (Pu *et al*., 2009).

### Network visualization

Network analysis and visualization were performed in Cytoscape (Smoot *et al*., 2010).

### Protein complex association

A cumulative hypergeometric probability was used to assign a *P*-value to the overrepresentation of complex members in the results of the genetic screen (Rivals *et al*., 2007). This test represents the probability that at least the same amount of protein members would be present in the screen when the same amount of genes identified in the screen were picked at random. It thus allows to identify protein complexes associated with colony morphology.

### Interactive network representation

An interactive version of the physical interaction network with the genes mapped from our genetic screen was developed using Cytoscape Web (Lopes *et al*., 2011).

### GO enrichment

GO enrichment was obtained through the BiNGO plugin (Maere *et al*., 2005) using a hypergeometric test and a Benjamini–Hochberg correction (Hochberg and Benjamini, 1990). GO annotations for *S. cerevisiae* were downloaded from the Gene Ontology (Ashburner *et al*., 2000) website (version 1.1600).

### Yeast strains

The whole genome screen was carried out using the Sigma 1278b deletion collection, a collection of 4156 strains, each of which carries a null mutation for one specific non-essential gene (Dowell *et al*., 2010). For an overview of all yeast strains used in this study see Table S6. Mutant strains were generated by amplifying the HygB cassette (pAG34) and the KANMX cassette (pUG6) from plasmids using primers (Table S7) that contained 60 bp sequence homologous to target DNA. The PCR product was then used for directed integration of the cassette and replacement of target locus. Yeast transformation was carried out using the LiAc procedure (Gietz and Woods, 2006). Transformants were verified by PCR using specific primers.

To obtain a series of mutants showing different levels of *FLO11* expression, we integrated a series of modified *TEF1* promoters (Nevoigt *et al*., 2006) directly upstream of the *FLO11* ORF. Strains overexpressing *TOS1* and *DFG16* were created by integrating a modified *TEF1* promoter (*TEF6* promoter from Nevoigt *et al*., 2006) directly upstream of the corresponding ORF.

To visualize Flo11 protein levels, we constructed a multicistronic DNA sequence encoding the *FLO11* gene, a viral self-cleaving peptide, and a gene encoding a yellow fluorescent protein (YFP). PCR transformation was used to incorporate the picornaviral 2A self-processing peptide sequence (de Felipe *et al*., 2006) at the 3′ end of the *FLO11* ORF. The 2A viral peptide sequence within the resulting *FLO11–*2A–YFP fusion allows for expression of multiple discrete proteins in equimolar quantities from a single transcript. The fusion construct thus generates a multicistronic mRNA from the *FLO11–*2A–YFP fusion, which is translated and thought to allow an intra-ribosomal cleavage event on the nascent protein to occur as the 2A peptide is exiting from the ribosome (de Felipe *et al*., 2006), and thus the two Flo11 and YFP proteins are produced separately. This method allows for a functional Flo11 protein to be expressed at the same time as the YFP so that we could monitor Flo11 expression without interfering with normal Flo11 function. Colony morphology phenotypes were retained in the *FLO11–*2A–YFP fusion constructs. Conventional fusions of YFP to *FLO11* interfered with Flo11 function, and abolished colony morphology phenotypes (data not shown).

Selected deletions (see Fig. S5) were created in the strain containing the *FLO11–*2A–YFP fusion construct. Yeast colonies grown for 5 days on solid media at 30°C were analysed by flow cytometry to quantify Flo11 expression levels.

### Growth assays

Yeast colonies were grown routinely for 5 days at 30°C unless otherwise noted. Yeast mats were grown on YPD or YPS with 0.3% agar for 14 days at room temperature. Colony morphology was assayed on YPS medium (2% agar), and colonies photographed using Nikon AZ100M with DS-R1 camera. Mat/colony area and height were measured with NIS Elements software and graphs were made in Prism with fitted curves.

### Desiccation experiments

Cells were plated from liquid YPD culture to form single colonies on a Nylon membrane (Millipore) placed on YPS solid medium and grown for 5 days at 30°C. After growth the membrane was removed and the colonies were placed in an empty Petri dish to dry for 8, 24 or 48h. To assess the number of dead cells within colonies, colonies were scraped off the plates and suspended in GM buffer (glucose 2%, Na-Hepes 10 mM, pH 7) and vortexed vigorously. Cells were stained with Live/Dead yeast viability stain (Invitrogen) with a final concentration of 20 μM and incubated for 30 min at 30°C in the dark. A Nikon TIE inverted scope equipped with a 60× oil objective, mCherry and GFP filter and a Luca R camera was used to determine the number of dead cells in biological triplicates. In all cases at least 300 cells were counted per sample per time point.

### Gene expression

Yeast colonies grown for 5 days on solid media at 30°C were harvested and frozen at −80°C in RNALater (Applied Biosystems) before processing for RNA extraction. RNA was extracted from cells by first spheroplasting the yeast cells for 1 h at 37°C using Solution A [Zymolyase, 1 mg ml^−1^ (MP Biomedicals); sorbitol, 0.9 M; EDTA pH 7.5, 0.1 M; β-mercaptoethanol, 14 mM] and subsequently using an ABI 6100 Nucleic Acid Prep Station and reagents (Applied Biosystems). Synthesis of cDNA was performed using the QuantiTect Reverse Transcription Kit (Qiagen). Real-time quantitative PCR (RT-PCR) was performed using the Power SYBR Green PCR Master Mix (Applied Biosystems). Analysis of *FLO11* transcript level was done using primers specific for *FLO11* and PCR reactions in a 25 μl volume in an Applied Biosystems StepOnePlus Real-Time OCR System and the following PCR program: 10 min at 95°C, followed by 40 cycles of 95°C for 15 s (melting), and 60°C for 1 min (annealing and extension). Expression values were normalized with levels of expression of a housekeeping gene (*ACT1*).

### Microarray

Yeast colonies were grown for 5 days on YPS at 30°C, harvested and frozen at −80°C before processing for RNA extraction. Total RNA was extracted using the hot phenol extraction method (Guthrie and Fink, 1991) and dissolved in 40 μl RNase free water. Quality control and array were performed by the VIB Micro Array Facility (http://www.microarray.be). The Affymetrix Yeast Genome 2.0 array was used for this experiment. This array contains probe sets to detect transcripts from both *S. cerevisiae* and *Schizosaccharomyces pombe*. This array includes approximately 5744 probe sets for 5841 of the 5845 genes present in *S. cerevisiae* and 5021 probe sets for all 5031 genes present in *S. pombe*. The sequence information for this array was selected by Affymetrix from the public data sources GenBankR (May 2004) and Sanger Center (June 2004) for the *S. cerevisiae* and *S. pombe* genomes respectively. These microarray data have been published in Gene Expression Omnibus under accession number GSE36151. The correlation between the RMA expression values for all samples was computed, and the intensities lower than the background signal (i.e. absent detection call) were omitted. The normalized intensity values over the different conditions were compared using the limma package (Smyth, 2004; Smyth *et al*., 2005) of the Bioconductor bioinformatics framework. For each of these contrasts, significant deviating values were selected using a moderated *t*-statistic, and additionally a Benjamini–Hochberg correction (Hochberg and Benjamini, 1990) was performed. Differentially expressed genes were selected based on the corrected *P*-values (*P* < 0.05) and a fold-change larger than 2 (log-ratio > 1) (Table S4). ClueGO (Bindea *et al*., 2009) was used to identify the biological processes, which were overrepresented in the differentially expressed genes between wild type and a *flo11* deletion mutant grown on liquid and solid media (Fig. S8). ClueGO was run as an Enrichment/Depletion (two-sided hypergeometric test) test with a Bonferroni correction for GO terms between level 3 and 8, a minimum of 8% of all genes in all groups and a kappa score threshold of 0.3. Finally, the identified GO terms were mapped onto our physical interaction network (Fig. 4).
