# Supplementary material for: Identification of a complex genetic network underlying Saccharomyces cerevisiae colony morphology
Source: Mol Microbiol. 2012 Sep 13;86(1):225–39. doi: 10.1111/j.1365-2958.2012.08192.x (PMC3470922; doi:10.1111/j.1365-2958.2012.08192.x)
Supplement: Supplementary file 1 [file mmi0086-0225-SD1.zip › mmi_8192_sm_FigureS1-8_TableS1-7.doc]

***Supplemental Information:***

**Identification of a complex genetic network underlying *Saccharomyces cerevisiae* colony morphology**

Karin Voordeckers^1,2*^, Dries De Maeyer^3*^, Elisa van der Zande^1,2*^, Marcelo D. Vinces^1,2*^, Wim Meert^1,2^, Lore Cloots³, Owen Ryan^4^, Kathleen Marchal^3,5^ and Kevin J. Verstrepen^1,2^

^1^ Laboratory for Systems Biology, VIB, Bio-Incubator, Gaston Geenslaan 1, B-3001 Leuven, Belgium.

^2^ Laboratory for Genetics and Genomics, Centre of Microbial and Plant Genetics (CMPG), KU Leuven, Gaston Geenslaan 1, B-3001 Leuven, Belgium.

Leuven, Belgium.

^3^ Department of Microbial and Molecular Systems, KU Leuven, Kasteelpark Arenberg 20, 3001 Leuven, Belgium

^4^ Banting and Best Department of Medical Research and Department of Molecular Genetics, Donnelly Centre, University of Toronto, 160 College St., Toronto, ON, Canada M5S 3E1

^5^ Department of Plant Biotechnology and Bioinformatics, Ghent University, Technologiepark 927, 9052 Gent, Belgium

* Equal contribution

Corresponding authors

Prof. Kevin Verstrepen

[Kevin.verstrepen@biw.vib-kuleuven.be](mailto:Kevin.verstrepen@biw.vib-kuleuven.be)

Prof. Kathleen Marchal

[kathleen.marchal@biw.kuleuven.be](https://owa.groupware.kuleuven.be/owa/redir.aspx?C=06834ae8414b48fdb3066e5125a6388a&URL=mailto%3Akathleen.marchal@biw.kuleuven.be)

Laboratory for Systems Biology

Gaston Geenslaan 1

3001 Leuven, Belgium

Tel: 0032 16 751 390

Fax: 0032 16 751 391

Running title: Genetic network for colony morphology in yeast.

Key words: yeast, genetic network, colony morphology, FLO11

**Fig. S1: Height vs area**

A	B

Colonies were grown on varying agar concentrations for 14 days at room temperature. The area of the colony was photographed with a Nikon AZ100 macroscope with 0.5X objective and measured with NIS Elements software. The height of the colonies was measured with the same macroscope with a 1X objective and Z-stacks were made. Analysis was done with NIS Elements.

**Fig. S2: Scoring of the genome wide screen.**

Normal morphology, no score

Smooth

Semi smooth

Extra wrinkly

Large

Small

No Growth

Weird

Based on these criteria the entire Sigma 1278b deletion collection was scored. Colonies with Smooth and Semi-Smooth morphologies were mapped in our genetic network.

**Fig. S3: GO enrichment of genes associated with altered colony morphology using GO's biological process ontology using a hypergeometric test with a Benjamini & Hochberg false discovery rate correction and a significance below 0.05.**

Genes associated with altered morphology mapped onto the physical interaction network and colored according to the associated biological process ontology GO terms based on lowest enrichment p-value, least overlap and level of the GO term. All nodes are genes identified in the genetic screening. Green edges indicate protein-protein interactions, blue edges phosphorylation interactions and red edges protein-DNA interactions. The width of an edge reflects the probability that the interaction exists based on experimental knowledge. Blue genes are associated with filamentous growth (GO:0030447), red genes with chromatin modification (GO:0006325), green with flocculation (GO:0000128) and biofilm formation (GO:0042710), orange with membrane invagination (GO:0010324), purple with ubiquitin-dependent protein catabolic process via the multivesicular body sorting pathway (GO:0043162) and dark grey with tRNA wobble uridine modification (GO:0002098).

**Fig. S4: KEGG pathway visualization of genes associated with altered colony morphology**

Genes associated with altered morphology mapped onto the physical interaction network and colored according to the associated biological process ontology GO terms based on lowest enrichment p-value, least overlap and level of the GO term. All nodes are genes identified in the genetic screening. Green edges indicate protein-protein interactions, blue edges phosphorylation interactions and red edges protein-DNA interactions. The width of an edge reflects the probability that the interaction exists based on experimental knowledge. Blue genes are associated with endocytosis (sce04144), red genes with sulphur relay (sce04122) and green with MAPK signaling pathways (sce04011).

**Fig. S5: Deletion mutants phenocopy colony morphology observed in genome-wide screen and this colony morphology phenotype is not linked to the marker gene used to create these deletions.**

A. Specific deletion strains (representing different processes identified as important for colony morphology) were created and subsequently grown in media with different carbon sources as described in the Experimental procedures. These strains phenocopy the result from the genome-wide screen. For *TOS1*, independently created deletion mutants displayed variable phenotypes, with three mutants having a wrinkly (shown here) and two having a semi-smooth colony morphology (the latter is the phenotype observed in screen). For more details, see main text. Glu, glucose; Mal, maltose; Gal, galactose; EtOH, ethanol; Gly, glycerol; Suc, sucrose.

B. Removal of marker gene does not affect colony morphology phenotype.

Mutants with (+) or without (-) the selection marker used to create these gene deletions show a similar phenotype when spotted on Ypsucrose.

**Fig. S6: Deletion mutants display altered Flo11 levels.**

Deletions were made in a strain carrying the self-cleavable *FLO11-2A-YFP* construct. Colonies of each deletion mutant were grown as described in the Experimental procedures and Flo11 levels were determined by flow cytometry. Four deletion mutants have reduced Flo11 levels, indicating that their effect on colony morphology is mainly mediated via changes in Flo11 expression. *Rvs161 (*encoding a cell raft protein involved in structural organization of the cell surface) and *arp8, sds3*, *swc7* and *rsc2* mutants (involved in chromatin structure and organization) do not display lower Flo11 levels, indicating that their effect might not be (completely) dependent on Flo11. For more details, see main text. Values represent data from three colonies per strain on YPS, relative to background fluorescence in WT strain grown on solid YPD. Error bars represent standard deviation.

**Fig. S7: Overexpression of *TOS1* and *DFG16* increases wrinkliness.**

Colonies overexpressing *TOS1* or *DFG16* are more wrinkly than WT strain. For details on strain construction, see Experimental procedures.

**Fig. S8: GO term enrichment/depletion between differentially expressed genes between *flo11* mutant and WT on solid and liquid media.**

Overview of GO term enrichment between *flo11* deletion mutant and WT grown on liquid and solid medium. Biological process GO terms are shown which are differentially expressed between mutant/WT on liquid and solid medium. The color and its intensity correspond to the medium in which the genes of the associated GO term are differentially expressed between the corresponding mutant, a green color indicates a high fraction of genes which are only expressed in liquid medium while the red color indicates genes only expressed in solid medium. The size of the node corresponds to the total numbers associated with this term. Terms associated with energy metabolism and mitochondrial processes can be seen in the grey areas.

**Table S1: Genome wide screen annotation, smooth and semi smooth morphologies**

| **ORFNAME** | **GENE NAME** | **BIOLOGICAL PROCESS** | **CODE** | **COLONY MORPHOLOGY ON YPS** |
| --- | --- | --- | --- | --- |
| YBR034C | HMT1 | mRNA export from nucleus* | 1 | small and smooth |
| YBR083W | TEC1 | pseudohyphal growth* | 1 | smooth |
| YBR105C | VID24 | vesicle-mediated transport* | 1 | semi smooth |
| YBR162C | TOS1 | biological process unknown | 1 | smooth and large |
| YBR222C | PCS60 | biological process unknown | 1 | smooth and large |
| YCL008C | STP22 | protein targeting to vacuole* | 1 | smooth and small |
| YCL018W | LEU2 | leucine biosynthesis | 1 | smooth and large |
| YCR094W | CDC50 |  | 1 | smooth and small |
| YDL233W |  | biological process unknown | 1 | large and smooth |
| YDR080W | VPS41 | homotypic vacuole fusion, non-autophagic* | 1 | small but wrinkly |
| YDR099W | BMH2 | sporulation (sensu Fungi)* | 1 | smooth and large |
| YDR387C |  | biological process unknown | 1 | semi-smooth |
| YEL007W |  | biological process unknown | 1 | smooth |
| YER027C | GAL83 | protein amino acid phosphorylation* | 1 | semi smooth |
| YER109C | FLO8 |  | 1 | semi smooth |
| YGL014W | PUF4 | mRNA catabolism, deadenylylation-dependent decay* | 1 | smooth |
| YGL045W | RIM8 |  | 1 | smooth and small |
| YGL068W | MNP1 |  | 1 | smooth and small |
| YGL211W | NCS6 | biological process unknown | 1 | semi smooth |
| YGR065C | VHT1 |  | 1 | smooth |
| YGR112W | SHY1 | aerobic respiration | 1 | small and wrinkly |
| YGR122W |  | biological process unknown | 1 | smooth |
| YGR208W | SER2 | serine family amino acid biosynthesis | 1 | smooth |
| YHL020C | OPI1 | positive regulation of transcription from RNA polymerase II promoter* | 1 | semi smooth and small |
| YHL027W | RIM101 | sporulation (sensu Fungi)* | 1 | smooth and large |
| YHL038C | CBP2 | Group I intron splicing | 1 | very little growth |
| YHR111W | UBA4 | pseudohyphal growth* | 1 | Semi Smooth |
| YIL063C | YRB2 |  | 1 | small and smooth |
| YIL134W | FLX1 | FAD transport | 1 | smooth |
| YJR102C | VPS25 | protein targeting to vacuole* | 1 | smooth |
| YLR025W | SNF7 | late endosome to vacuole transport* | 1 | smooth and small |
| YLR131C | ACE2 | G1-specific transcription in mitotic cell cycle | 1 | smooth and large |
| YLR154C | RNH203 | DNA replication | 1 | smooth |
| YLR396C | VPS33 |  | 1 | smooth |
| YLR417W | VPS36 | protein targeting to vacuole* | 1 | smooth |
| YML055W | SPC2 | signal peptide processing | 1 | could be contamination |
| YMR044W | IOC4 | chromatin remodeling | 1 | semi smooth |
| YMR077C | VPS20 | late endosome to vacuole transport* | 1 | smooth |
| YMR154C | RIM13 | protein processing | 1 | smooth and small |
| YMR231W | PEP5 | late endosome to vacuole transport* | 1 | Smooth + Yellow |
| YNL084C | END3 | endocytosis* | 1 |  |
| YNL183C | NPR1 | regulation of nitrogen utilization | 1 | smooth and relatively small |
| YOL068C | HST1 | chromatin silencing | 1 | smooth |
| YOL086C | ADH1 |  | 1 | small and smooth |
| *YOL140W* | *ARG8* |  | 1 | smooth and small |
| YOR027W | STI1 | protein folding | 1 | smooth and small |
| YOR030W | DFG16 | invasive growth (sensu Saccharomyces)* | 1 | smooth |
| YOR183W | FYV12 |  | 1 | smooth and large |
| YOR369C | RPS12 |  | 1 | small and smooth |
| YPL002C | SNF8 | protein targeting to vacuole* | 1 | flat |
| YPL262W | FUM1 | tricarboxylic acid cycle* | 1 | very small smooth colony |
| YPR040W | TIP41 | signal transduction | 1 | small and smooth |
| YAL005C | SSA1 |  | 2 | smooth |
| YAL037W |  | biological process unknown | 2 | wrinkly |
| YBL002W | HTB2 | chromatin assembly or disassembly | 2 | semi smooth |
| YBL020W | RFT1 |  | 2 | semi smooth |
| YBL029W |  | biological process unknown | 2 | semi smooth |
| YBL058W | SHP1 | sporulation (sensu Fungi)* | 2 | small and smooth |
| YBL071W-A | KTI11 | G1 phase of mitotic cell cycle* | 2 | small and semi smooth |
| YBR081C | SPT7 |  | 2 | semi smooth |
| YBR111W-A | SUS1 | mRNA export from nucleus* | 2 | wrinkly |
| YBR158W | AMN1 | negative regulation of exit from mitosis* | 2 | semi smooth |
| YBR165W | UBS1 | protein export from nucleus* | 2 | semi smooth |
| YBR214W | SDS24 | endocytosis* | 2 | semi smooth |
| YBR233W-A | DAD3 |  | 2 | semi smooth |
| YBR281C |  | biological process unknown | 2 | semi smooth |
| YCR009C | RVS161 | endocytosis* | 2 | semi smooth |
| YDL001W | RMD1 | biological process unknown | 2 | semi smooth |
| YDL004W | ATP16 |  | 2 | semi smooth and small |
| YDL040C | NAT1 |  | 2 | small and semi smooth |
| YDL047W | SIT4 |  | 2 | semi smooth |
| YDL090C | RAM1 | protein amino acid farnesylation | 2 | wrinkly |
| YDL146W | LDB17 |  | 2 | smooth and small |
| YDL191W | RPL35A | protein biosynthesis | 2 | small and wrinkly |
| YDR006C | SOK1 | cAMP-mediated signaling | 2 | semi smooth |
| YDR079C-A | TFB5 | DNA repair* | 2 | small but wrinkly |
| YDR090C |  | biological process unknown | 2 | semi smooth |
| YDR105C | TMS1 | biological process unknown | 2 | large and semi-smooth |
| YDR134C |  | biological process unknown | 2 | semi smooth |
| YDR137W | RGP1 | intracellular protein transport | 2 | wrinkly but wetter |
| YDR159W | SAC3 | mRNA export from nucleus* | 2 | small but wrinkly |
| YDR161W |  | biological process unknown | 2 | small but wrinkly |
| YDR162C | NBP2 | response to heat* | 2 | small but wrinkly |
| YDR174W | HMO1 | plasmid maintenance | 2 | semi smooth |
| YDR184C | ATC1 | response to stress* | 2 | semi smooth |
| YDR205W | MSC2 | zinc ion homeostasis | 2 | semi smooth |
| YDR213W | UPC2 | sterol biosynthesis* | 2 | wrinkly |
| YDR266C |  | biological process unknown | 2 | normal wrinklyness; but smaller |
| YDR276C | PMP3 | cation transport | 2 | semi smooth |
| YDR289C | RTT103 | transcription termination from Pol II promoter, RNA polymerase(A) coupled* | 2 | normal wrinklyness; but smaller |
| YDR292C | SRP101 |  | 2 | semi-smooth |
| YDR320C | SWA2 | ER organization and biogenesis | 2 | normal wrinklyness; but smaller |
| YDR388W | RVS167 | endocytosis* | 2 | semi smooth |
| YDR418W | RPL12B | protein biosynthesis* | 2 | small but wrinkly |
| YDR448W | ADA2 | histone acetylation* | 2 | small but wrinkly |
| YDR484W | VPS52 | Golgi to vacuole transport* | 2 | small but wrinkly |
| YDR486C | VPS60 | filamentous growth* | 2 | semi smooth |
| YDR511W | ACN9 | gluconeogenesis* | 2 | small but wrinkly |
| YDR512C | EMI1 | sporulation (sensu Fungi) | 2 | small but wrinkly |
| YEL013W | VAC8 | protein targeting to vacuole* | 2 | small but wrinkly |
| YEL025C |  |  | 2 | semi smooth |
| YEL053C | MAK10 | N-terminal protein amino acid acetylation | 2 | semi-smooth |
| YER019W | ISC1 | response to salt stress* | 2 | semi-smooth |
| YER020W | GPA2 | sporulation (sensu Fungi)* | 2 | semi-smooth |
| YER056C-A | RPL34A | protein biosynthesis | 2 | semi_smooth |
| YER089C | PTC2 | G1/S transition of mitotic cell cycle* | 2 | maybe due to contaminant |
| YER118C | SHO1 | pseudohyphal growth* | 2 | semi smooth |
| YFL060C | SNO3 |  | 2 | semi smooth |
| YGL003C | CDH1 | mitotic sister chromatid segregation* | 2 | only on edges |
| YGL167C | PMR1 | secretory pathway* | 2 | semi smooth |
| YGL168W | HUR1 | DNA replication | 2 | semi smooth |
| YGL228W | SHE10 | biological process unknown | 2 | semi smooth |
| YGR123C | PPT1 | protein amino acid dephosphorylation | 2 | semi smooth |
| YGR168C |  | biological process unknown | 2 | semi smooth |
| YGR180C | RNR4 | DNA replication | 2 | semi smooth |
| YGR181W | TIM13 | protein import into mitochondrial inner membrane | 2 | semi smooth |
| YGR197C | SNG1 | response to drug | 2 | semi smooth |
| YGR229C | SMI1 | cell wall organization and biogenesis* | 2 | small and semi smooth |
| YGR250C |  | biological process unknown | 2 | semi smooth |
| YGR266W |  | biological process unknown | 2 | large and smooth |
| YHL006C | SHU1 | error-free DNA repair | 2 | semi smooth |
| YHL007C | STE20 | protein amino acid phosphorylation* | 2 | semi smooth and small |
| YHR013C | ARD1 | protein amino acid acetylation | 2 | semi-smooth |
| YHR071W | PCL5 |  | 2 | semi smooth and small |
| YHR082C | KSP1 | protein amino acid phosphorylation | 2 | semi smooth |
| YHR084W | STE12 | pseudohyphal growth* | 2 | semi smooth and small |
| YHR114W | BZZ1 | actin filament organization* | 2 | semi smooth |
| YHR119W | SET1 | chromatin silencing at telomere* | 2 | smooth sector |
| YHR124W | NDT80 | meiosis* | 2 | wrinkly |
| YHR167W | THP2 | mRNA export from nucleus* | 2 | wrinkly |
| YHR168W | MTG2 | protein biosynthesis | 2 | NG |
| YHR204W | MNL1 | ER-associated protein catabolism | 2 |  |
| YIL006W |  | transport* | 2 | semi smooth |
| YIL008W | URM1 | response to oxidative stress* | 2 | Semi Smooth, normal size |
| YIL014C-A |  | biological process unknown | 2 | smooth sectors |
| YIL084C | SDS3 | chromatin silencing* | 2 | small and semi smooth |
| YIL101C | XBP1 | response to stress | 2 | semi_smooth |
| YIL118W | RHO3 |  | 2 | smooth |
| YIL153W | RRD1 | DNA repair* | 2 | small and wrinkly |
| YIL166C |  | transport | 2 | semi smooth |
| YIL167W | SDL1 | serine family amino acid metabolism | 2 | semi smooth |
| YIR019C | FLO11/MUC1 | pseudohyphal growth* | 2 | semi smooth |
| YJL046W |  | biological process unknown | 2 | semi_smooth |
| YJL088W | ARG3 |  | 2 | semi_smooth |
| YJL189W | RPL39 |  | 2 | semi smooth and small |
| YJL208C | NUC1 | DNA recombination* | 2 | semi_smooth |
| YJR001W | AVT1 | neutral amino acid transport | 2 | semi smooth |
| YJR101W | RSM26 |  | 2 | semi smooth and small |
| YKL038W | RGT1 | glucose metabolism* | 2 | smaller and smoother |
| YKL039W | PTM1 | biological process unknown | 2 | wrinkly and normal size |
| YKL051W | SFK1 | actin cytoskeleton organization and biogenesis* | 2 | some smooth sectors |
| YKL062W | MSN4 | response to stress* | 2 | some smooth sectors |
| YKL064W | MNR2 | magnesium ion transport | 2 | wrinkly and normal size |
| YKL072W | STB6 | biological process unknown | 2 | semi smooth |
| YKL114C | APN1 | DNA repair* | 2 | crater like wrinkly |
| YKL185W | ASH1 | pseudohyphal growth* | 2 | semi smooth |
| YLL042C | ATG10 | protein targeting to vacuole* | 2 | semi smooth |
| YLR052W | IES3 | chromatin remodeling | 2 | semi smooth and large |
| YLR061W | RPL22A | protein biosynthesis | 2 | small and wrinkly |
| YLR099W-A |  | biological process unknown | 2 | semi smooth |
| YLR337C | VRP1 | endocytosis* | 2 | semi smooth |
| YLR357W | RSC2 | chromatin remodeling* | 2 | semi-smooth and large |
| YLR370C | ARC18 | actin filament organization* | 2 | small and wrinkly |
| YLR385C | SWC7 | chromatin remodeling | 2 | semi-smooth |
| YLR390W-A | CCW14 | cell wall organization and biogenesis | 2 | small and semi smooth |
| YLR408C |  | biological process unknown | 2 |  |
| YLR418C | CDC73 | RNA elongation from RNA polymerase II promoter* | 2 | semi smooth |
| YLR435W | TSR2 | processing of 20S pre-rRNA | 2 | small and wrinkly |
| YLR442C | SIR3 |  | 2 | semi smooth |
| YML035C | AMD1 |  | 2 | semi smooth |
| YML073C | RPL6A | protein biosynthesis* | 2 | small and wrinkly |
| YML081C-A | ATP18 | ATP synthesis coupled proton transport | 2 | small and wrinkly |
| YMR063W | RIM9 | sporulation (sensu Fungi) | 2 | semi smooth |
| YMR135C | GID8 |  | 2 | semi smooth |
| YMR164C | MSS11 | pseudohyphal growth* | 2 | smooth and large |
| YNL079C | TPM1 | actin filament organization* | 2 | slightly less wrinkly |
| YNL082W | PMS1 | meiosis* | 2 | semi-smooth |
| YNL119W | NCS2 | pseudohyphal growth* | 2 | semi smooth and small |
| YNL121C | TOM70 | protein import into mitochondrial matrix* | 2 | semi smooth and small |
| YNL210W | MER1 |  | 2 | semi smooth |
| YNL294C | RIM21 | sporulation (sensu Fungi)* | 2 | semi smooth |
| YNR052C | POP2 |  | 2 | semi smooth |
| YOL004W | SIN3 | regulation of transcription from RNA polymerase II promoter* | 2 | small colony, less wrinkly |
| YOL045W | PSK2 | protein amino acid phosphorylation* | 2 | semi smooth |
| YOL047C |  |  | 2 | semi_smooth |
| YOL049W | GSH2 |  | 2 | semi smooth |
| YOL076W | MDM20 | mitochondrion inheritance* | 2 | small and semi smooth |
| YOR025W | HST3 | chromatin silencing at telomere* | 2 | semi smooth |
| YOR081C | TGL5 | triacylglycerol mobilization | 2 | semi smooth |
| YOR136W | IDH2 | tricarboxylic acid cycle* | 2 | semi smooth and small |
| YOR141C | ARP8 | biological process unknown | 2 | small and semi smooth |
| YOR165W | SEY1 | membrane organization and biogenesis | 2 | smooth edge |
| YOR267C | HRK1 | cell ion homeostasis | 2 | smooth edges |
| YOR275C | RIM20 | invasive growth (sensu Saccharomyces)* | 2 | smooth with one crater |
| YOR375C | GDH1 | glutamate biosynthesis | 2 | nice double wrinkliness |
| YPL019C | VTC3 | vacuole fusion, non-autophagic | 2 | little bit wrinkly in the middel |
| YPL051W | ARL3 | intracellular protein transport | 2 | small and semi smooth |
| YPL058C | PDR12 | transport* | 2 | semi smooth and smaller |
| YPL077C |  | biological process unknown | 2 | semi smooth, large |
| YPL084W | BRO1 | ubiquitin-dependent protein catabolism* | 2 | larger colony, semi-smooth |
| YPL099C | FMP14 | biological process unknown | 2 | semi smooth |
| YPL101W | ELP4 | regulation of transcription from RNA polymerase II promoter | 2 | semi smooth, small |
| YPL112C | PEX25 | peroxisome organization and biogenesis* | 2 | smooth edges |
| YPL158C |  | biological process unknown | 2 | smaller colony, semi-smooth |
| YPL184C |  | biological process unknown | 2 | semi smooth |
| YPL250C | ICY2 | biological process unknown | 2 | smooth edges |
| YPL256C | CLN2 | regulation of cyclin dependent protein kinase activity* | 2 | smooth edges |
| YPR067W | ISA2 |  | 2 | semi smooth and small |
| YPR070W | MED1 | transcription from RNA polymerase II promoter | 2 | super wrinkly |
| YPR172W |  | biological process unknown | 2 | smooth edges |
| YPR189W | SKI3 | mRNA catabolism* | 2 | semi smooth |

**Table S2:** Cumulative hypergeometric probabilities for overrepresentation of protein complex members in genetic screening data.

| **Complex name** | **Cumulative hypergeometric probability** |
| --- | --- |
| Rvs161p/Rvs167p complex | <1E-16 |
| ESCRT II complex | <1E-16 |
| HOPS complex | 2,19E-05 |
| NatA complex | 4,42E-05 |
| Tap42p/Sit4p/Rrd1p complex | 4,42E-05 |
| ESCRT III complex | 0,000172102 |
| Rpd3L complex | 0,000611511 |
| CORVET complex | 0,000816002 |
| Sap190p/Sit4p complex | 0,001255613 |
| guanyl-nucleotide exchange factor complex | 0,001255613 |
| Tim8p/Tim13p complex | 0,001255613 |
| Car1p/Arg3p complex | 0,001255613 |
| Sap155p/Sit4p complex | 0,001255613 |
| Mlh1p/Pms1p complex | 0,001255613 |
| Sap185p/Sit4p complex | 0,001255613 |
| Sac3p/Thp1p complex | 0,001255613 |
| Cdc28p/Cln2p complex | 0,001255613 |
| NatB complex | 0,001255613 |
| Isocitrate dehydrogenase | 0,001255613 |
| protein farnesyltransferase complex | 0,001255613 |
| Cdc42p/Ste20p complex | 0,001255613 |
| signal recognition particle receptor complex | 0,001255613 |
| FBP degradation complex | 0,002167019 |
| ribonuclease H2 complex | 0,003678466 |
| Dig1p/Ste12p/Dig2p complex | 0,003678466 |
| NatC complex | 0,003678466 |
| ribonucleoside-diphosphate reductase complex | 0,003678466 |
| GSH degradosomal complex | 0,003678466 |
| ISW1b complex | 0,003678466 |
| Sum1p/Rfm1p/Hst1p complex | 0,003678466 |
| Ski Complex | 0,003678466 |
| chromatin silencing complex | 0,003678466 |
| actin cytoskeleton-regulatory complex, Pan1p/Sla1p/End3p complex | 0,003678466 |
| Snf1p/Snf4p/Gal83p complex | 0,003678466 |
| RNA Degradation Complex | 0,003678466 |
| SAGA complex | 0,004787051 |
| VTC heterotetrameric complex | 0,007184829 |
| ESCRT I complex | 0,007184829 |
| GARP complex | 0,007184829 |
| THO complex | 0,007184829 |
| signal peptidase complex | 0,007184829 |
| Ino80p complex | 0,007662428 |
| Rpd3S complex | 0,011695401 |
| Ada2p/Gcn5p/Ada3 transcription activator complex | 0,011695401 |
| Elongator complex | 0,017135101 |
| SLIK (SAGA-like) complex | 0,020785178 |
| Set3p complex | 0,023432871 |
| mitochondrial outer membrane translocase complex | 0,023432871 |
| Arp2/3 protein complex | 0,023432871 |
| Cdc73p/Paf1p complex | 0,023432871 |
| F0/F1 ATP synthase (complex V) | 0,024302927 |
| cytoplasmic ribosomal large subunit | 0,024313662 |
| COMPASS complex | 0,030521489 |
| Nucleosomal protein complex | 0,030521489 |
| CCR4-NOT core complex | 0,038337385 |
| transcription factor TFIIH complex | 0,046820471 |
| DASH complex | 0,046820471 |
| Swr1p complex | 0,075720793 |
| anaphase-promoting complex | 0,097364148 |

**Table S3: Overview of enrichments in shaded areas in Figure 2 (overview genetic screening). GO terms for both biological process and cellular component where calculated for all genes found in genetic screening with a corrected p-value lower than 0.20 and a Benjamini & Hochberg false discovery rate correction. If more than 33% of all genes in a GO term enrichment set could be associated with a shaded area the term was associated with this area. GO terms associated with regulation were not omitted. Additionally p-values for associated protein complexes larger than size 2 were added to this table.**

| GO_ID | Description | p-value | | Corrected p-value | x | n | X | N |
| --- | --- | --- | --- | --- | --- | --- | --- | --- |
|  | | | | | | | | |
| **RIM101** | | | | | | | | |
|  |  |  | |  |  |  |  |  |
| *Biological process* | | | | | | | | |
| 43162 | ubiquitin-dependent protein catabolic process via the multivesicular body sorting pathway | 4.66E-06 | | 2.56E-04 | 7 | 22 | 211 | 6320 |
| 7034 | vacuolar transport | 1.35E-05 | | 5.87E-04 | 16 | 139 | 211 | 6320 |
| 71453 | cellular response to oxygen levels | 1.16E-03 | | 0.0191 | 3 | 7 | 211 | 6320 |
| 45324 | late endosome to vacuole transport | 1.09E-03 | | 0.0191 | 6 | 36 | 211 | 6320 |
| 16485 | protein processing | 1.67E-03 | | 0.025816 | 6 | 39 | 211 | 6320 |
| 6623 | protein targeting to vacuole | 1.82E-03 | | 0.026484 | 9 | 84 | 211 | 6320 |
| 9268 | response to pH | 1.81E-03 | | 0.026484 | 3 | 8 | 211 | 6320 |
| 70482 | response to oxygen levels | 1.81E-03 | | 0.026484 | 3 | 8 | 211 | 6320 |
| 16197 | endosome transport | 3.93E-03 | | 0.043219 | 8 | 77 | 211 | 6320 |
| 44265 | cellular macromolecule catabolic process | 3.90E-03 | | 0.043219 | 21 | 336 | 211 | 6320 |
| 51604 | protein maturation | 4.41E-03 | | 0.046918 | 6 | 47 | 211 | 6320 |
| 31667 | response to nutrient levels | 5.57E-03 | | 0.057292 | 9 | 99 | 211 | 6320 |
| 71454 | cellular response to anoxia | 6.37E-03 | | 0.062207 | 2 | 4 | 211 | 6320 |
| 34059 | response to anoxia | 6.37E-03 | | 0.062207 | 2 | 4 | 211 | 6320 |
| 9057 | macromolecule catabolic process | 7.07E-03 | | 0.065474 | 21 | 354 | 211 | 6320 |
| 9605 | response to external stimulus | 7.20E-03 | | 0.065561 | 9 | 103 | 211 | 6320 |
| 9991 | response to extracellular stimulus | 7.20E-03 | | 0.065561 | 9 | 103 | 211 | 6320 |
| 70676 | intralumenal vesicle formation | 1.04E-02 | | 0.084707 | 2 | 5 | 211 | 6320 |
| 7584 | response to nutrient | 1.32E-02 | | 0.099837 | 4 | 28 | 211 | 6320 |
| 6511 | ubiquitin-dependent protein catabolic process | 2.85E-02 | | 0.16726 | 12 | 194 | 211 | 6320 |
| 19941 | modification-dependent protein catabolic process | 2.95E-02 | | 0.16819 | 12 | 195 | 211 | 6320 |
| 70481 | nuclear-transcribed mRNA catabolic process, non-stop decay | 3.13E-02 | | 0.17042 | 3 | 21 | 211 | 6320 |
| 31669 | cellular response to nutrient levels | 3.70E-02 | | 0.18005 | 7 | 94 | 211 | 6320 |
| 51603 | proteolysis involved in cellular protein catabolic process | 4.23E-02 | | 0.19261 | 12 | 206 | 211 | 6320 |
|  |  |  | |  |  |  |  |  |
| *Cellular component* | | | | | | | | |
| 44440 | endosomal part | 5.55E-06 | | 0.001605 | 13 | 88 | 211 | 6347 |
| 5768 | endosome | 1.16E-05 | | 0.001681 | 16 | 138 | 211 | 6347 |
| 814 | ESCRT II complex | 3.62E-05 | | 0.003491 | 3 | 3 | 211 | 6347 |
| 10008 | endosome membrane | 7.43E-05 | | 0.003604 | 11 | 82 | 211 | 6347 |
| 815 | ESCRT III complex | 6.31E-03 | | 0.11526 | 2 | 4 | 211 | 6347 |
| 9898 | internal side of plasma membrane | 1.47E-02 | | 0.16013 | 3 | 16 | 211 | 6347 |
|  |  |  | |  |  |  |  |  |
| *Protein complexes* | | | | | | | | |
|  | ESCRT II complex | 0.00E+00 | |  |  |  |  |  |
|  | ESCRT III complex | 1.72E-04 | |  |  |  |  |  |
|  | ESCRT I complex | 7.18E-03 | |  |  |  |  |  |
|  |  |  | |  |  |  |  |  |
|  |  |  | |  |  |  |  |  |
| **tRNA modification** | | | | | | | | |
|  |  | |  |  |  |  |  |  |
| *Biological process* | | |  |  |  |  |  |  |
| 2143 | wobble position uridine thiolation | | 5.88E-06 | 3.10E-04 | 4 | 5 | 211 | 6320 |
| 2098 | tRNA wobble uridine modification | | 1.60E-05 | 6.54E-04 | 7 | 26 | 211 | 6320 |
| 2097 | tRNA wobble base modification | | 1.60E-05 | 6.54E-04 | 7 | 26 | 211 | 6320 |
| 32447 | protein urmylation | | 3.91E-05 | 0.001074 | 4 | 7 | 211 | 6320 |
| 34227 | tRNA thio-modification | | 3.91E-05 | 0.001074 | 4 | 7 | 211 | 6320 |
| 6400 | tRNA modification | | 6.65E-03 | 0.062817 | 7 | 67 | 211 | 6320 |
| 19954 | asexual reproduction | | 3.44E-02 | 0.17042 | 6 | 73 | 211 | 6320 |
| 7114 | cell budding | | 3.44E-02 | 0.17042 | 6 | 73 | 211 | 6320 |
|  |  | |  |  |  |  |  |  |
|  |  | |  |  |  |  |  |  |
| **Chromatin remodelling** | | |  |  |  |  |  |  |
|  |  | |  |  |  |  |  |  |
| *Biological process* | | |  |  |  |  |  |  |
| 43162 | ubiquitin-dependent protein catabolic process via the multivesicular body sorting pathway | | 4.66E-06 | 2.56E-04 | 7 | 22 | 211 | 6320 |
| 6350 | transcription | | 3.27E-05 | 0.00106 | 37 | 558 | 211 | 6320 |
| 6351 | transcription, DNA-dependent | | 3.27E-05 | 0.00106 | 37 | 558 | 211 | 6320 |
| 32774 | RNA biosynthetic process | | 3.40E-05 | 0.001074 | 37 | 559 | 211 | 6320 |
| 16568 | chromatin modification | | 9.08E-05 | 0.002252 | 18 | 197 | 211 | 6320 |
| 6325 | chromatin organization | | 1.49E-04 | 0.003317 | 20 | 242 | 211 | 6320 |
| 6623 | protein targeting to vacuole | | 1.82E-03 | 0.026484 | 9 | 84 | 211 | 6320 |
| 34605 | cellular response to heat | | 2.44E-03 | 0.032162 | 5 | 29 | 211 | 6320 |
| 16575 | histone deacetylation | | 3.84E-03 | 0.042991 | 4 | 20 | 211 | 6320 |
| 9408 | response to heat | | 4.41E-03 | 0.046918 | 6 | 47 | 211 | 6320 |
| 31667 | response to nutrient levels | | 5.57E-03 | 0.057292 | 9 | 99 | 211 | 6320 |
| 9266 | response to temperature stimulus | | 6.01E-03 | 0.060853 | 6 | 50 | 211 | 6320 |
| 6476 | protein amino acid deacetylation | | 6.49E-03 | 0.062207 | 4 | 23 | 211 | 6320 |
| 9605 | response to external stimulus | | 7.20E-03 | 0.065561 | 9 | 103 | 211 | 6320 |
| 9991 | response to extracellular stimulus | | 7.20E-03 | 0.065561 | 9 | 103 | 211 | 6320 |
| 16569 | covalent chromatin modification | | 1.07E-02 | 0.085689 | 8 | 91 | 211 | 6320 |
| 16570 | histone modification | | 1.07E-02 | 0.085689 | 8 | 91 | 211 | 6320 |
| 7584 | response to nutrient | | 1.32E-02 | 0.099837 | 4 | 28 | 211 | 6320 |
| 51276 | chromosome organization | | 1.31E-02 | 0.099837 | 23 | 422 | 211 | 6320 |
| 6338 | chromatin remodeling | | 1.39E-02 | 0.10482 | 7 | 77 | 211 | 6320 |
| 11 | vacuole inheritance | | 2.07E-02 | 0.13529 | 3 | 18 | 211 | 6320 |
| 6368 | RNA elongation from RNA polymerase II promoter | | 2.88E-02 | 0.16726 | 5 | 52 | 211 | 6320 |
| 8298 | intracellular mRNA localization | | 3.54E-02 | 0.17354 | 3 | 22 | 211 | 6320 |
| 31669 | cellular response to nutrient levels | | 3.70E-02 | 0.18005 | 7 | 94 | 211 | 6320 |
| 6342 | chromatin silencing | | 4.08E-02 | 0.19109 | 7 | 96 | 211 | 6320 |
| 6354 | RNA elongation | | 4.07E-02 | 0.19109 | 5 | 57 | 211 | 6320 |
|  |  | |  |  |  |  |  |  |
| *Cellular component* | | |  |  |  |  |  |  |
| 814 | ESCRT II complex | | 3.62E-05 | 0.003491 | 3 | 3 | 211 | 6347 |
| 16585 | chromatin remodeling complex | | 7.34E-04 | 0.026513 | 11 | 106 | 211 | 6347 |
| 45239 | tricarboxylic acid cycle enzyme complex | | 3.23E-03 | 0.084804 | 2 | 3 | 211 | 6347 |
| 785 | chromatin | | 9.92E-03 | 0.14332 | 10 | 128 | 211 | 6347 |
| 790 | nuclear chromatin | | 1.13E-02 | 0.14779 | 9 | 111 | 211 | 6347 |
| 33698 | Rpd3L complex | | 1.47E-02 | 0.16013 | 3 | 16 | 211 | 6347 |
| 70822 | Sin3-type complex | | 2.04E-02 | 0.19929 | 3 | 18 | 211 | 6347 |
|  |  | |  |  |  |  |  |  |
| *Protein complexes* | | |  |  |  |  |  |  |
|  | Rpd3L complex | | 6.12E-04 |  |  |  |  |  |
|  | SAGA complex | | 4.79E-03 |  |  |  |  |  |
|  | SLIK (SAGA-like) complex | | 2.08E-02 |  |  |  |  |  |
|  | Rpd3S complex | | 1.17E-02 |  |  |  |  |  |
|  | Swr1p complex | | 7.57E-02 |  |  |  |  |  |
|  | Ino80p complex | | 7.66E-03 |  |  |  |  |  |
|  |  | |  |  |  |  |  |  |
|  |  | |  |  |  |  |  |  |
| **Endocytosis** | | |  |  |  |  |  |  |
|  |  | |  |  |  |  |  |  |
| *Biological process* | | |  |  |  |  |  |  |
| 16044 | cellular membrane organization | | 3.87E-05 | 0.001074 | 23 | 275 | 211 | 6320 |
| 7033 | vacuole organization | | 3.78E-05 | 0.001074 | 10 | 63 | 211 | 6320 |
| 61024 | membrane organization | | 3.87E-05 | 0.001074 | 23 | 275 | 211 | 6320 |
| 10324 | membrane invagination | | 3.52E-05 | 0.001074 | 15 | 134 | 211 | 6320 |
| 30029 | actin filament-based process | | 8.23E-04 | 0.01577 | 11 | 107 | 211 | 6320 |
| 6897 | endocytosis | | 1.08E-03 | 0.0191 | 10 | 94 | 211 | 6320 |
| 51666 | actin cortical patch localization | | 1.23E-03 | 0.020004 | 4 | 15 | 211 | 6320 |
| 42144 | vacuole fusion, non-autophagic | | 2.44E-03 | 0.032162 | 5 | 29 | 211 | 6320 |
| 16192 | vesicle-mediated transport | | 2.72E-03 | 0.033794 | 23 | 370 | 211 | 6320 |
| 16237 | microautophagy | | 2.80E-03 | 0.034375 | 6 | 43 | 211 | 6320 |
| 51469 | vesicle fusion with vacuole | | 3.26E-03 | 0.037566 | 2 | 3 | 211 | 6320 |
| 60988 | lipid tube assembly | | 3.26E-03 | 0.037566 | 2 | 3 | 211 | 6320 |
| 34727 | piecemeal microautophagy of nucleus | | 4.38E-03 | 0.046918 | 5 | 33 | 211 | 6320 |
| 31667 | response to nutrient levels | | 5.57E-03 | 0.057292 | 9 | 99 | 211 | 6320 |
| 30036 | actin cytoskeleton organization | | 6.77E-03 | 0.063391 | 9 | 102 | 211 | 6320 |
| 9605 | response to external stimulus | | 7.20E-03 | 0.065561 | 9 | 103 | 211 | 6320 |
| 9991 | response to extracellular stimulus | | 7.20E-03 | 0.065561 | 9 | 103 | 211 | 6320 |
| 7010 | cytoskeleton organization | | 1.93E-02 | 0.12958 | 13 | 205 | 211 | 6320 |
| 11 | vacuole inheritance | | 2.07E-02 | 0.13529 | 3 | 18 | 211 | 6320 |
| 9651 | response to salt stress | | 2.40E-02 | 0.14928 | 3 | 19 | 211 | 6320 |
| 6591 | ornithine metabolic process | | 2.72E-02 | 0.16308 | 2 | 8 | 211 | 6320 |
| 6944 | cellular membrane fusion | | 2.87E-02 | 0.16726 | 6 | 70 | 211 | 6320 |
| 61025 | membrane fusion | | 2.87E-02 | 0.16726 | 6 | 70 | 211 | 6320 |
| 71255 | CVT vesicle assembly | | 3.34E-02 | 0.17042 | 1 | 1 | 211 | 6320 |
| 71562 | nucleus-vacuole junction assembly | | 3.34E-02 | 0.17042 | 1 | 1 | 211 | 6320 |
| 6611 | protein export from nucleus | | 3.13E-02 | 0.17042 | 3 | 21 | 211 | 6320 |
| 19954 | asexual reproduction | | 3.44E-02 | 0.17042 | 6 | 73 | 211 | 6320 |
| 22614 | membrane to membrane docking | | 3.34E-02 | 0.17042 | 1 | 1 | 211 | 6320 |
| 7114 | cell budding | | 3.44E-02 | 0.17042 | 6 | 73 | 211 | 6320 |
| 7015 | actin filament organization | | 3.56E-02 | 0.17404 | 5 | 55 | 211 | 6320 |
| 6914 | autophagy | | 4.08E-02 | 0.19109 | 7 | 96 | 211 | 6320 |
|  |  | |  |  |  |  |  |  |
| *Cellular component* | | |  |  |  |  |  |  |
| 30897 | HOPS complex | | 6.73E-04 | 0.026513 | 3 | 6 | 211 | 6347 |
| 329 | fungal-type vacuole membrane | | 6.38E-03 | 0.11526 | 10 | 120 | 211 | 6347 |
| 43332 | mating projection tip | | 7.93E-03 | 0.13487 | 9 | 105 | 211 | 6347 |
| 44463 | cell projection part | | 1.06E-02 | 0.14637 | 9 | 110 | 211 | 6347 |
| 5937 | mating projection | | 1.55E-02 | 0.16013 | 9 | 117 | 211 | 6347 |
| 42995 | cell projection | | 1.55E-02 | 0.16013 | 9 | 117 | 211 | 6347 |
| 33263 | CORVET complex | | 1.51E-02 | 0.16013 | 2 | 6 | 211 | 6347 |
|  |  | |  |  |  |  |  |  |
| Protein complexes | | |  |  |  |  |  |  |
|  | Rvs161p/Rvs167p complex | | 0.00E+00 |  |  |  |  |  |
|  | HOPS complex | | 2.19E-05 |  |  |  |  |  |
|  | CORVET complex | | 8.16E-04 |  |  |  |  |  |
|  | actin cytoskeleton-regulatory complex, Pan1p/Sla1p/End3p complex | | 3.68E-03 |  |  |  |  |  |
|  |  | |  |  |  |  |  |  |
|  |  | |  |  |  |  |  |  |
| **MAPK** |  | |  |  |  |  |  |  |
|  |  | |  |  |  |  |  |  |
| *Biological process* | | |  |  |  |  |  |  |
| 9628 | response to abiotic stimulus | | 1.95E-04 | 0.004186 | 13 | 122 | 211 | 6320 |
| 6970 | response to osmotic stress | | 6.59E-04 | 0.013452 | 9 | 73 | 211 | 6320 |
| 34605 | cellular response to heat | | 2.44E-03 | 0.032162 | 5 | 29 | 211 | 6320 |
| 1402 | signal transduction involved in filamentous growth | | 3.70E-03 | 0.04217 | 3 | 10 | 211 | 6320 |
| 9408 | response to heat | | 4.41E-03 | 0.046918 | 6 | 47 | 211 | 6320 |
| 9266 | response to temperature stimulus | | 6.01E-03 | 0.060853 | 6 | 50 | 211 | 6320 |
| 70887 | cellular response to chemical stimulus | | 6.49E-03 | 0.062207 | 16 | 241 | 211 | 6320 |
| 71470 | cellular response to osmotic stress | | 1.16E-02 | 0.091093 | 4 | 27 | 211 | 6320 |
| 71214 | cellular response to abiotic stimulus | | 1.49E-02 | 0.10883 | 4 | 29 | 211 | 6320 |
| 11 | vacuole inheritance | | 2.07E-02 | 0.13529 | 3 | 18 | 211 | 6320 |
| 51704 | multi-organism process | | 2.46E-02 | 0.15237 | 9 | 126 | 211 | 6320 |
| 7231 | osmosensory signaling pathway | | 3.54E-02 | 0.17354 | 3 | 22 | 211 | 6320 |
| 23014 | signal transmission via phosphorylation event | | 3.97E-02 | 0.18966 | 3 | 23 | 211 | 6320 |
| 7243 | intracellular protein kinase cascade | | 3.97E-02 | 0.18966 | 3 | 23 | 211 | 6320 |
| 22610 | biological adhesion | | 4.18E-02 | 0.19109 | 2 | 10 | 211 | 6320 |
| 7155 | cell adhesion | | 4.18E-02 | 0.19109 | 2 | 10 | 211 | 6320 |
|  |  | |  |  |  |  |  |  |
| *Protein complexes* | | |  |  |  |  |  |  |
|  | Snf1p/Snf4p/Gal83p complex | | 3.68E-03 |  |  |  |  |  |
|  | Tap42p/Sit4p/Rrd1p complex | | 4.42E-05 |  |  |  |  |  |
|  |  | |  |  |  |  |  |  |
|  |  | |  |  |  |  |  |  |
| **Not assigned** | | |  |  |  |  |  |  |
|  |  | |  |  |  |  |  |  |
| *Biological process* | | |  |  |  |  |  |  |
| 6402 | mRNA catabolic process | | 2.71E-02 | 0.16308 | 7 | 88 | 211 | 6320 |
| 46907 | intracellular transport | | 5.34E-03 | 0.055866 | 33 | 628 | 211 | 6320 |
| 7124 | pseudohyphal growth | | 1.78E-05 | 7.05E-04 | 10 | 58 | 211 | 6320 |
| 34204 | lipid translocation | | 3.42E-02 | 0.17042 | 2 | 9 | 211 | 6320 |
| 10961 | cellular magnesium ion homeostasis | | 3.34E-02 | 0.17042 | 1 | 1 | 211 | 6320 |
| 16049 | cell growth | | 2.82E-05 | 9.66E-04 | 10 | 61 | 211 | 6320 |
| 32505 | reproduction of a single-celled organism | | 8.05E-03 | 0.070957 | 14 | 204 | 211 | 6320 |
| 15031 | protein transport | | 1.14E-02 | 0.090908 | 27 | 513 | 211 | 6320 |
| 9056 | catabolic process | | 2.95E-02 | 0.16819 | 35 | 762 | 211 | 6320 |
| 43934 | sporulation | | 9.13E-04 | 0.016979 | 14 | 161 | 211 | 6320 |
| 18193 | peptidyl-amino acid modification | | 7.39E-04 | 0.014387 | 7 | 46 | 211 | 6320 |
| 31365 | N-terminal protein amino acid modification | | 3.31E-04 | 0.006871 | 4 | 11 | 211 | 6320 |
| 45184 | establishment of protein localization | | 1.42E-02 | 0.10561 | 27 | 522 | 211 | 6320 |
| 7568 | aging | | 2.37E-02 | 0.14845 | 6 | 67 | 211 | 6320 |
| 9084 | glutamine family amino acid biosynthetic process | | 1.49E-02 | 0.10883 | 4 | 29 | 211 | 6320 |
| 30437 | ascospore formation | | 7.66E-03 | 0.068762 | 9 | 104 | 211 | 6320 |
| 22402 | cell cycle process | | 3.51E-02 | 0.17335 | 25 | 514 | 211 | 6320 |
| 6810 | transport | | 3.15E-03 | 0.037239 | 57 | 1215 | 211 | 6320 |
| 30435 | sporulation resulting in formation of a cellular spore | | 8.58E-04 | 0.016204 | 14 | 160 | 211 | 6320 |
| 32502 | developmental process | | 1.26E-02 | 0.097239 | 19 | 327 | 211 | 6320 |
| 9653 | anatomical structure morphogenesis | | 2.94E-02 | 0.16819 | 14 | 240 | 211 | 6320 |
| 128 | flocculation | | 1.16E-03 | 0.0191 | 3 | 7 | 211 | 6320 |
| 43412 | macromolecule modification | | 1.42E-02 | 0.10561 | 36 | 748 | 211 | 6320 |
| 6464 | protein modification process | | 1.43E-03 | 0.022923 | 34 | 602 | 211 | 6320 |
| 501 | flocculation via cell wall protein-carbohydrate interaction | | 1.52E-02 | 0.10883 | 2 | 6 | 211 | 6320 |
| 1304 | progressive alteration of chromatin involved in replicative cell aging | | 2.72E-02 | 0.16308 | 2 | 8 | 211 | 6320 |
| 34613 | cellular protein localization | | 1.68E-02 | 0.11642 | 21 | 384 | 211 | 6320 |
| 48856 | anatomical structure development | | 2.94E-02 | 0.16819 | 14 | 240 | 211 | 6320 |
| 70727 | cellular macromolecule localization | | 7.73E-03 | 0.068905 | 23 | 403 | 211 | 6320 |
| 51716 | cellular response to stimulus | | 5.67E-03 | 0.057805 | 34 | 655 | 211 | 6320 |
| 7569 | cell aging | | 2.37E-02 | 0.14845 | 6 | 67 | 211 | 6320 |
| 48646 | anatomical structure formation involved in morphogenesis | | 7.09E-03 | 0.065474 | 14 | 201 | 211 | 6320 |
| 9607 | response to biotic stimulus | | 3.09E-02 | 0.17042 | 4 | 36 | 211 | 6320 |
| 1301 | progressive alteration of chromatin involved in cell aging | | 4.18E-02 | 0.19109 | 2 | 10 | 211 | 6320 |
| 51649 | establishment of localization in cell | | 1.45E-03 | 0.022972 | 37 | 674 | 211 | 6320 |
| 1403 | invasive growth in response to glucose limitation | | 1.66E-07 | 4.19E-05 | 11 | 45 | 211 | 6320 |
| 9987 | cellular process | | 4.10E-02 | 0.19109 | 180 | 5088 | 211 | 6320 |
| 51641 | cellular localization | | 1.90E-03 | 0.027059 | 41 | 782 | 211 | 6320 |
| 6950 | response to stress | | 2.67E-03 | 0.033419 | 38 | 722 | 211 | 6320 |
| 40007 | growth | | 5.28E-08 | 1.67E-05 | 18 | 118 | 211 | 6320 |
| 6345 | loss of chromatin silencing | | 2.09E-02 | 0.13529 | 2 | 7 | 211 | 6320 |
| 6473 | protein amino acid acetylation | | 2.88E-02 | 0.16726 | 5 | 52 | 211 | 6320 |
| 6996 | organelle organization | | 2.00E-03 | 0.028065 | 59 | 1244 | 211 | 6320 |
| 19935 | cyclic-nucleotide-mediated signaling | | 4.18E-02 | 0.19109 | 2 | 10 | 211 | 6320 |
| 22413 | reproductive process in single-celled organism | | 3.77E-02 | 0.1825 | 9 | 136 | 211 | 6320 |
| 51234 | establishment of localization | | 1.85E-03 | 0.026531 | 59 | 1240 | 211 | 6320 |
| 43687 | post-translational protein modification | | 5.47E-03 | 0.056735 | 26 | 461 | 211 | 6320 |
| 42710 | biofilm formation | | 6.81E-04 | 0.013676 | 3 | 6 | 211 | 6320 |
| 34203 | glycolipid translocation | | 3.34E-02 | 0.17042 | 1 | 1 | 211 | 6320 |
| 44182 | filamentous growth of a population of unicellular organisms | | 1.68E-08 | 7.10E-06 | 15 | 75 | 211 | 6320 |
| 956 | nuclear-transcribed mRNA catabolic process | | 2.56E-02 | 0.15815 | 7 | 87 | 211 | 6320 |
| 51179 | localization | | 4.49E-03 | 0.047352 | 61 | 1341 | 211 | 6320 |
| 6605 | protein targeting | | 2.53E-03 | 0.032973 | 18 | 260 | 211 | 6320 |
| 10960 | magnesium ion homeostasis | | 3.34E-02 | 0.17042 | 1 | 1 | 211 | 6320 |
| 48468 | cell development | | 7.66E-03 | 0.068762 | 9 | 104 | 211 | 6320 |
| 46836 | glycolipid transport | | 3.34E-02 | 0.17042 | 1 | 1 | 211 | 6320 |
| 30447 | filamentous growth | | 7.07E-10 | 8.95E-07 | 18 | 91 | 211 | 6320 |
| 3 | reproduction | | 1.94E-02 | 0.13009 | 19 | 342 | 211 | 6320 |
| 43935 | sexual sporulation resulting in formation of a cellular spore | | 1.03E-02 | 0.084707 | 9 | 109 | 211 | 6320 |
| 6474 | N-terminal protein amino acid acetylation | | 1.33E-04 | 0.003029 | 4 | 9 | 211 | 6320 |
| 18409 | peptide or protein amino-terminal blocking | | 3.31E-04 | 0.006871 | 4 | 11 | 211 | 6320 |
| 44248 | cellular catabolic process | | 4.24E-03 | 0.046282 | 34 | 643 | 211 | 6320 |
| 51301 | cell division | | 1.07E-02 | 0.085689 | 18 | 299 | 211 | 6320 |
| 45039 | protein import into mitochondrial inner membrane | | 3.42E-02 | 0.17042 | 2 | 9 | 211 | 6320 |
| 33036 | macromolecule localization | | 1.80E-02 | 0.12168 | 34 | 709 | 211 | 6320 |
| 1302 | replicative cell aging | | 1.78E-02 | 0.1213 | 5 | 46 | 211 | 6320 |
| 19953 | sexual reproduction | | 1.03E-02 | 0.084707 | 14 | 210 | 211 | 6320 |
| 48308 | organelle inheritance | | 6.63E-03 | 0.062817 | 6 | 51 | 211 | 6320 |
| 1308 | loss of chromatin silencing involved in replicative cell aging | | 2.09E-02 | 0.13529 | 2 | 7 | 211 | 6320 |
| 50896 | response to stimulus | | 2.04E-03 | 0.028355 | 47 | 935 | 211 | 6320 |
| 6401 | RNA catabolic process | | 8.65E-03 | 0.074458 | 9 | 106 | 211 | 6320 |
| 6886 | intracellular protein transport | | 3.04E-03 | 0.036464 | 21 | 329 | 211 | 6320 |
| 30154 | cell differentiation | | 3.75E-03 | 0.042411 | 14 | 187 | 211 | 6320 |
| 34293 | sexual sporulation | | 1.03E-02 | 0.084707 | 9 | 109 | 211 | 6320 |
| 8104 | protein localization | | 2.29E-02 | 0.14635 | 29 | 594 | 211 | 6320 |
| 19933 | cAMP-mediated signaling | | 4.18E-02 | 0.19109 | 2 | 10 | 211 | 6320 |
| 71033 | nuclear retention of pre-mRNA at the site of transcription | | 3.42E-02 | 0.17042 | 2 | 9 | 211 | 6320 |
| 70783 | growth of unicellular organism as a thread of attached cells | | 1.68E-08 | 7.10E-06 | 15 | 75 | 211 | 6320 |
| 6850 | mitochondrial pyruvate transport | | 3.34E-02 | 0.17042 | 1 | 1 | 211 | 6320 |
| 16043 | cellular component organization | | 3.41E-02 | 0.17042 | 72 | 1786 | 211 | 6320 |
| 15878 | biotin transport | | 3.34E-02 | 0.17042 | 1 | 1 | 211 | 6320 |
|  |  | |  |  |  |  |  |  |
| *Cellular function* | | |  |  |  |  |  |  |
| 70390 | transcription export complex 2 | | 6.31E-03 | 0.11526 | 2 | 4 | 211 | 6347 |
| 31248 | protein acetyltransferase complex | | 7.48E-05 | 0.003604 | 4 | 8 | 211 | 6347 |
| 43234 | protein complex | | 2.13E-03 | 0.068385 | 63 | 1359 | 211 | 6347 |
| 43229 | intracellular organelle | | 9.34E-03 | 0.14203 | 158 | 4272 | 211 | 6347 |
| 43226 | organelle | | 9.34E-03 | 0.14203 | 158 | 4272 | 211 | 6347 |
| 31414 | N-terminal protein acetyltransferase complex | | 7.48E-05 | 0.003604 | 4 | 8 | 211 | 6347 |
| 31415 | NatA complex | | 3.23E-03 | 0.084804 | 2 | 3 | 211 | 6347 |
| 44464 | cell part | | 4.98E-03 | 0.11061 | 202 | 5766 | 211 | 6347 |
| 5623 | cell | | 4.98E-03 | 0.11061 | 202 | 5766 | 211 | 6347 |
| 5737 | cytoplasm | | 1.34E-02 | 0.16013 | 152 | 4109 | 211 | 6347 |
| 34657 | GID complex | | 2.07E-02 | 0.19929 | 2 | 7 | 211 | 6347 |

**Table S4: Differentially expressed genes in *flo11* mutant versus WT in liquid and solid medium (see also Supplemental File S1)**

**Differentially expressed in liquid medium**

YHR140W, YFR015C, YEL011W, YKL003C, YIL134W, YCR046C, LR371W, YPR133W-A, YMR120C, YER058W, YAL044C, YHR116W, YBR091C, YLR209C, YPL172C, YLR109W, YLL043W, YPR003C, YKL163W, YLR135W, YJR045C, YOR079C, YCR071C, YDR178W, YIL070C, YJL046W, YLR102C, YNR037C, YDR041W, YLR423C, YML030W, YBR120C, YDL171C, YLR072W, YJL096W, YLR273C, YOR065W, YMR286W, YPR160W, YLR431C, YDR256C, YBR044C, YLL040C, YNL073W, YOR176W, YKL040C, YKL148C, YHL038C, YPL040C, YDR237W, YLR436C, YER182W, YLR128W, YGL068W, YMR012W, YJR034W, YLR265C, YGR142W, YDR079W, YLL022C, YDR540C, YLR375W, YJR048W, YBR122C, YLR318W, YOR071C, YLR214W, YOR187W, YLR089C, YHR136C, YIL162W, YKR091W, YKL084W, YDL184C, YLR097C, YGR286C, YMR105C, YLR212C, YHR059W, YLR404W, YDR515W, YFR033C, YLR254C, YGR055W, YLR427W, YBR251W, YLR141W, YLR100W, YBL015W, YJL133C-A, YBL059C-A, YLL003W, YJL063C, YLR345W, YML129C, YBL090W, YLR053C, YGR220C, YDR316W, YLR456W, YNR022C, YNL280C, YLR246W, YLR407W, YDL205C, YIL098C, YNL315C, YLR251W, YER037W, YLR025W, YOR266W, YBR185C, YNR060W, YNL066W, YKR039W, YKR093W, YGL262W, YML120C, YLR361C, YLR447C, YGR150C, YAR066W, YKL170W, YER044C-A, YLR413W, YLR049C, YBR146W, YLR271W, YPL189C-A, YDL124W, YNL185C, YJR133W, YLR430W, YHL004W, YGR121C, YPL132W, YLR297W, YLR360W, YLR005W, YBR282W, YMR173W, YOR358W, YLL018C-A, YIL093C, YHL048W, YOR389W, YGR174C, YJR016C, JL208C, YLR088W, YGR215W, YNL306W, YLR328W, YLR337C, YLR228C, YGL037C, YHR179W, YLR095C, YMR098C, YLR021W, YDR019C, YLR211C, YOL071W, YDR430C, YCL064C, YEL024W, YDR268W, YNL284C, YLR066W, YAL038W, YBR296C, YJL062W-A, YNL119W, YNL081C, YLR050C, YGR181W, YLR451W, YLR343W, YLR258W, YDR232W, YDR322W, YLR130C, YMR158W, YLR300W, YAR071W, YLR281C, YLL012W, YFL016C, YBL059W, YLL060C, YLR178C, YIL121W, YBR268W, YLR126C, YJL200C, YKL138C, YOR356W, YLR177W, YCR003W, YHL033C, YLR046C, YLR011W, YLR274W, YLR368W, YGR084C, YLR213C, YER156C, YBR214W, YDR375C, YKR066C, YFR007W, YPL215W, YLR288C, YMR323W, YKL167C, YEL050C, YJR135W-A, YLR078C, YDR258C, YOR196C, YBR037C, YDR534C, YER174C, YGL129C, YOR177C, YEL070W, YOR384W, YGR165W, YLL054C, YLR353W, YDR337W, YLR315W, YPL201C, YDR493W, YLR414C, YML025C, YLR154C, YLR306W, YLR001C, YKL087C, YAL046C, YLR460C, YPL059W, YLR290C, YLL049W, YMR193W, YDL120W, YKL194C, YML050W, YDL045W-A, YNL122C, YBR022W, YLL021W, YKR085C, YLL038C, YKL134C, YER093C-A, YMR319C, YPL104W, YDR234W, YOR045W, YNL100W, YPL183W-A, YNL177C, YKL137W, YDR393W, YGL263W, YKL161C, YFL065C, YGL236C, YLR256W, YLR417W, YOL155C, YJR115W, YHR038W, YPR100W, YMR322C, YLR166C, YLR433C, YKL152C, YLL063C, YLR396C, YPR166C, YGL009C, YLR181C, YLR093C, YLR263W, YNL005C, YMR228W, YMR282C, YLR056W, YLR085C, YLR399C, YJL104W, YPL277C, YNR067C, YDL202W, YLR248W, YPL252C, YDR116C, YLR224W, YHR015W, YKR006C, YLL031C, YBR006W, YLR018C, YLR450W, YLR189C, YJL088W, YDR194C, YMR024W, YMR225C, YLR350W, YPL173W, YML128C, YNR040W, YDL021W, YNL083W, YOL023W, YDR175C, YJR097W, YER170W, YOR158W, YFR045W, YCR104W, YGR060W, YLR376C, YLL048C, YLR313C, YMR188C, YGL028C, YHR037W, YDR347W, YLR410W, YDR381C-A, YDL039C, YLR192C, YKL191W, YGR029W, YLL058W, YNL070W, YGL143C, YLR352W

**Differentially expressed in both liquid and solid medium**

YLR324W, YPL187W, YKL209C, YLR074C, YPR015C, YNL234W, YLR064W, YLR131C, YLR442C, YLR243W, YLL053C, YLR121C, YOR388C, YOR315W, YOR348C, YIL015W, YJL157C, YJR005C-A, YKL178C, YLR136C, YLR030W, YNL146C-A, YLR270W, YLR362W, YDR461W, YHL047C, YCR097W, YLL028W, YHL024W, YHR087W, YLR381W, YLR137W, YOL152W, YOR313C, YLR183C, YJL170C, YJR004C, YLR108C, YDR536W, YLR080W, YLR298C, YJL116C, YLR047C, YLR449W, YGR248W, YLR446W, YER124C, YLR036C, YLR245C, YLL056C, YLR206W, YLR278C, YCL066W, YLR412W, YLR022C, YLL051C, YLR316C, YIL099W, YLR219W, YLR045C, YLR040C, YLR164W, YLR120C, YLR034C, YHR143W, YLR031W, YGL209W, YLR443W, YFL026W, YLR016C, YLR356W, YMR189W, YCL027W, YKL141W, YLL010C, YLR299W, YLL015W, YGL089C, YOR161C, YLL023C, YLR142W, YJR122W, YLR149C, YGL032C, YLR054C, YLR459W, YNR044W, YHR209W, YJR153W, YIR019C, YLR151C, YLR052W, YNR002C, YLR387C, YGR213C, YLR286C, YLR194C, YLL055W, YNL145W, YLR125W, YLR454W, YGR249W, YLR260W, YLL052C

**Differentially expressed in solid medium**

YEL057C, YPL095C, YGL240W, YBL075C, YLR332W, YLR051C, YLR090W, YLR309C, YLR342W, YOL047C, YLL034C, YDR277C, YLR455W, YML047C, YOR047C, YLR363W-A, YIL119C, YPL058C, YLR267W, YPR111W, YER095W, YOL147C, YLL008W, YLR180W, YJL214W, YBR238C, YDL222C, YLR285W, YHR092C, YLR287C, YDR223W, YLR094C, YLR069C, YLR197W, YDR184C, YDL245C, YLR218C, YGR211W, YGR109W-A, YGR023W, YLR015W, YPL054W, YMR230W-A, YMR169C, YLR227C, YLR409C, YKR034W, YLR435W, YLR019W, YOL048C, YPL130W, YOL158C, YLR405W, YAR020C, YPL250C, YLR132C, YLR225C, YLL013C, YFL054C, YLR092W, YCL038C, YKR075C, YNL194C, YLR203C, YLL029W, YER028C, YOL084W, YLR010C, YGL253W, YDL087C, YLR127C, YPL151C, YLR196W, YLL018C, YAL053W, YDR285W, YLR002C, YDR336W, YLR032W, YLR083C, YFL020C, YPR121W, YLL046C, YDL048C, YLL062C, YLR008C, YMR095C, YOR382W, YLR009W, YJL144W, YHR033W, YDL204W, YJL045W, YGR109W-B, YDL241W, YLL032C, YCL054W, YJR149W, YML083C, YMR013W-A, YGR177C, YLR323C, YKL051W, YLR222C, YMR084W, YLR438W, YGL183C, YKL218C, YEL066W, YOR338W, YHR048W, YAL061W, YLR129W, YKR041W, YPL089C, YHL022C, YLR073C, YJR019C, YLR401C, YLR175W, YOR306C, YLR346C, YOL002C, YGL256W, YLR138W, YPR002W, YLR226W, YLR107W, YPL207W, YOR212W, YLR234W, YPL222W, YLR303W, YLR347C, YLR133W, YLR042C, YLR139C, YPL150W, YBL081W, YDL223C, YLR284C, YLL061W, YOL058W, YLR429W, YNL279W, YLL011W, YLR272C, YNR050C, YLR113W, YGL035C, YCR020C, YEL073C, YLL057C, YMR011W, YGR239C, YLR012C, YDR056C, YNR034W, YCR045C, YDR213W, YLR004C, YPL088W, YLR276C, YLR146C, YIL045W, YML058W-A, YLL036C, YLR421C, YEL072W, YHL043W, YLR173W, YBL086C, YOL151W, YOR383C, YLR116W, YGR144W, YLR033W, YDL085C-A, YLR336C, YLR119W, YLR357W, YOR204W, YLR392C, YLR221C, YOR349W, YJL056C, YPL036W, YLR223C, YLR055C, YGL255W, YLR329W, YOL156W, YLL035W, YLR262C, YFL053W, YLL024C, YDR034W-B, YIL014C-A, YMR251W, YLR186W, YCR096C, YGL230C, YBR240C, YIL066C, YOL154W, YJL087C

**Table S5: Overview of enrichments in shaded areas in Figure 4 (overview microarray experiments). GO terms for both biological process and cellular component where calculated for all differentially expressed genes between the *flo11* mutant and WT in both liquid and solid medium with a corrected p-value lower than 0.30 and a Benjamini & Hochberg false discovery rate correction. If more than 33% of all genes in a GO term enrichment set could be associated with a shaded area the term was associated with this area. GO terms associated with regulation were omitted.**

| GO_ID | Description | p-value | Corrected p-value | x | n | X | N |
| --- | --- | --- | --- | --- | --- | --- | --- |
|  | |  |  |  |  |  |  |
| **Ion homeostasis** | |  |  |  |  |  |  |
|  |  |  |  |  |  |  |  |
| *Biological process* | |  |  |  |  |  |  |
| 55072 | iron ion homeostasis | 1.90E-07 | 6.93E-05 | 19 | 50 | 651 | 6320 |
| 55066 | di-, tri-valent inorganic cation homeostasis | 2.00E-07 | 6.93E-05 | 25 | 80 | 651 | 6320 |
| 22900 | electron transport chain | 1.35E-05 | 0.002931 | 19 | 64 | 651 | 6320 |
| 55080 | cation homeostasis | 1.58E-05 | 0.003036 | 29 | 124 | 651 | 6320 |
| 48878 | chemical homeostasis | 3.00E-05 | 0.00473 | 31 | 141 | 651 | 6320 |
| 50801 | ion homeostasis | 3.77E-05 | 0.00544 | 30 | 136 | 651 | 6320 |
| 15891 | siderophore transport | 7.47E-05 | 0.009241 | 6 | 9 | 651 | 6320 |
| 30005 | cellular di-, tri-valent inorganic cation homeostasis | 2.67E-04 | 0.025653 | 17 | 66 | 651 | 6320 |
| 6121 | mitochondrial electron transport, succinate to ubiquinone | 5.13E-04 | 0.038594 | 4 | 5 | 651 | 6320 |
| 6879 | cellular iron ion homeostasis | 6.86E-04 | 0.045686 | 11 | 36 | 651 | 6320 |
| 6811 | ion transport | 7.26E-04 | 0.046553 | 33 | 181 | 651 | 6320 |
| 42592 | homeostatic process | 8.71E-04 | 0.053897 | 40 | 235 | 651 | 6320 |
| 6779 | porphyrin biosynthetic process | 3.78E-03 | 0.10876 | 6 | 16 | 651 | 6320 |
| 6783 | heme biosynthetic process | 3.78E-03 | 0.10876 | 6 | 16 | 651 | 6320 |
| 33014 | tetrapyrrole biosynthetic process | 3.78E-03 | 0.10876 | 6 | 16 | 651 | 6320 |
| 6778 | porphyrin metabolic process | 5.34E-03 | 0.12331 | 6 | 17 | 651 | 6320 |
| 33013 | tetrapyrrole metabolic process | 5.34E-03 | 0.12331 | 6 | 17 | 651 | 6320 |
| 42168 | heme metabolic process | 5.34E-03 | 0.12331 | 6 | 17 | 651 | 6320 |
| 15677 | copper ion import | 5.56E-03 | 0.12677 | 4 | 8 | 651 | 6320 |
| 30001 | metal ion transport | 5.75E-03 | 0.12868 | 15 | 72 | 651 | 6320 |
| 30003 | cellular cation homeostasis | 5.80E-03 | 0.12868 | 20 | 107 | 651 | 6320 |
| 41 | transition metal ion transport | 6.07E-03 | 0.13147 | 10 | 40 | 651 | 6320 |
| 6873 | cellular ion homeostasis | 9.33E-03 | 0.17004 | 21 | 119 | 651 | 6320 |
| 55082 | cellular chemical homeostasis | 1.02E-02 | 0.17646 | 21 | 120 | 651 | 6320 |
| 35434 | copper ion transmembrane transport | 1.41E-02 | 0.21621 | 4 | 10 | 651 | 6320 |
| 6826 | iron ion transport | 1.42E-02 | 0.21648 | 5 | 15 | 651 | 6320 |
| 6825 | copper ion transport | 1.90E-02 | 0.26765 | 5 | 16 | 651 | 6320 |
|  |  |  |  |  |  |  |  |
| *Cellular component* | |  |  |  |  |  |  |
| 5749 | mitochondrial respiratory chain complex II | 5.07E-04 | 0.005296 | 4 | 5 | 652 | 6347 |
| 45257 | succinate dehydrogenase complex (ubiquinone) | 5.07E-04 | 0.005296 | 4 | 5 | 652 | 6347 |
| 45283 | fumarate reductase complex | 5.07E-04 | 0.005296 | 4 | 5 | 652 | 6347 |
| 45281 | succinate dehydrogenase complex | 1.40E-03 | 0.013472 | 4 | 6 | 652 | 6347 |
| 45273 | respiratory chain complex II | 1.40E-03 | 0.013472 | 4 | 6 | 652 | 6347 |
|  |  |  |  |  |  |  |  |
|  |  |  |  |  |  |  |  |
| **Glycogen biosynthesis** | | |  |  |  |  |  |
|  |  |  |  |  |  |  |  |
| *Biological process* | |  |  |  |  |  |  |
| 5978 | glycogen biosynthetic process | 4.88E-03 | 0.11904 | 5 | 12 | 651 | 6320 |
| 44042 | glucan metabolic process | 1.30E-02 | 0.20726 | 9 | 38 | 651 | 6320 |
| 6073 | cellular glucan metabolic process | 1.30E-02 | 0.20726 | 9 | 38 | 651 | 6320 |
| 9250 | glucan biosynthetic process | 1.64E-02 | 0.24474 | 6 | 21 | 651 | 6320 |
| 5977 | glycogen metabolic process | 1.69E-02 | 0.24944 | 7 | 27 | 651 | 6320 |
|  |  |  |  |  |  |  |  |
|  |  |  |  |  |  |  |  |
| **Thiamin biosynthesis** | | |  |  |  |  |  |
|  |  |  |  |  |  |  |  |
| *Biological process* | |  |  |  |  |  |  |
| 6772 | thiamin metabolic process | 1.90E-02 | 0.26765 | 5 | 16 | 651 | 6320 |
|  |  |  |  |  |  |  |  |
|  |  |  |  |  |  |  |  |
| **RNA processing** | |  |  |  |  |  |  |
|  |  |  |  |  |  |  |  |
| *Cellular component* | |  |  |  |  |  |  |
| 34388 | Pwp2p-containing subcomplex of 90S preribosome | 1.70E-02 | 0.13057 | 3 | 6 | 652 | 6347 |
|  |  |  |  |  |  |  |  |
|  |  |  |  |  |  |  |  |
| **Monosaccharide transport** | | |  |  |  |  |  |
|  |  |  |  |  |  |  |  |
| *Biological process* | |  |  |  |  |  |  |
| 15749 | monosaccharide transport | 6.68E-03 | 0.1412 | 7 | 23 | 651 | 6320 |
| 8645 | hexose transport | 6.68E-03 | 0.1412 | 7 | 23 | 651 | 6320 |
|  |  |  |  |  |  |  |  |
|  |  |  |  |  |  |  |  |
| **Mitochondrial translation** | | |  |  |  |  |  |
|  |  |  |  |  |  |  |  |
| *Biological process* | |  |  |  |  |  |  |
| 7005 | mitochondrion organization | 4.08E-33 | 7.07E-30 | 109 | 317 | 651 | 6320 |
| 32543 | mitochondrial translation | 1.19E-32 | 1.03E-29 | 64 | 120 | 651 | 6320 |
| 6996 | organelle organization | 5.14E-05 | 0.006847 | 167 | 1244 | 651 | 6320 |
| 70127 | tRNA aminoacylation for mitochondrial protein translation | 7.27E-03 | 0.14806 | 5 | 13 | 651 | 6320 |
| 70124 | mitochondrial translational initiation | 9.28E-03 | 0.17004 | 3 | 5 | 651 | 6320 |
| 6422 | aspartyl-tRNA aminoacylation | 1.06E-02 | 0.17646 | 2 | 2 | 651 | 6320 |
|  |  |  |  |  |  |  |  |
| *Cellular component* | |  |  |  |  |  |  |
| 313 | organellar ribosome | 1.25E-38 | 2.35E-36 | 58 | 84 | 652 | 6347 |
| 5761 | mitochondrial ribosome | 1.25E-38 | 2.35E-36 | 58 | 84 | 652 | 6347 |
| 5759 | mitochondrial matrix | 1.27E-37 | 1.20E-35 | 92 | 211 | 652 | 6347 |
| 31980 | mitochondrial lumen | 1.27E-37 | 1.20E-35 | 92 | 211 | 652 | 6347 |
| 44429 | mitochondrial part | 2.17E-31 | 1.63E-29 | 152 | 577 | 652 | 6347 |
| 315 | organellar large ribosomal subunit | 7.49E-23 | 4.02E-21 | 32 | 44 | 652 | 6347 |
| 5762 | mitochondrial large ribosomal subunit | 7.49E-23 | 4.02E-21 | 32 | 44 | 652 | 6347 |
| 5739 | mitochondrion | 8.32E-20 | 3.91E-18 | 208 | 1146 | 652 | 6347 |
| 5763 | mitochondrial small ribosomal subunit | 2.16E-14 | 8.12E-13 | 22 | 34 | 652 | 6347 |
| 314 | organellar small ribosomal subunit | 2.16E-14 | 8.12E-13 | 22 | 34 | 652 | 6347 |
| 33279 | ribosomal subunit | 4.23E-10 | 1.45E-08 | 56 | 232 | 652 | 6347 |
| 5840 | ribosome | 2.51E-09 | 7.86E-08 | 61 | 275 | 652 | 6347 |
| 15934 | large ribosomal subunit | 3.67E-07 | 8.11E-06 | 34 | 134 | 652 | 6347 |
| 31974 | membrane-enclosed lumen | 5.29E-07 | 1.10E-05 | 139 | 924 | 652 | 6347 |
| 70013 | intracellular organelle lumen | 3.85E-05 | 5.17E-04 | 125 | 878 | 652 | 6347 |
| 43233 | organelle lumen | 3.85E-05 | 5.17E-04 | 125 | 878 | 652 | 6347 |
| 15935 | small ribosomal subunit | 2.98E-04 | 0.003418 | 22 | 98 | 652 | 6347 |
| 30529 | ribonucleoprotein complex | 8.88E-03 | 0.075895 | 85 | 649 | 652 | 6347 |
|  |  |  |  |  |  |  |  |
|  |  |  |  |  |  |  |  |
| **Electron transport chain** | | |  |  |  |  |  |
|  |  |  |  |  |  |  |  |
| *Biological process* | |  |  |  |  |  |  |
| 22900 | electron transport chain | 1.35E-05 | 0.002931 | 19 | 64 | 651 | 6320 |
| 6121 | mitochondrial electron transport, succinate to ubiquinone | 5.13E-04 | 0.038594 | 4 | 5 | 651 | 6320 |
| 42773 | ATP synthesis coupled electron transport | 1.41E-03 | 0.066947 | 9 | 28 | 651 | 6320 |
| 42775 | mitochondrial ATP synthesis coupled electron transport | 1.41E-03 | 0.066947 | 9 | 28 | 651 | 6320 |
| 45333 | cellular respiration | 2.04E-03 | 0.088387 | 21 | 105 | 651 | 6320 |
| 22904 | respiratory electron transport chain | 3.10E-03 | 0.10552 | 9 | 31 | 651 | 6320 |
| 9060 | aerobic respiration | 3.22E-03 | 0.10725 | 18 | 88 | 651 | 6320 |
| 6779 | porphyrin biosynthetic process | 3.78E-03 | 0.10876 | 6 | 16 | 651 | 6320 |
| 6783 | heme biosynthetic process | 3.78E-03 | 0.10876 | 6 | 16 | 651 | 6320 |
| 33014 | tetrapyrrole biosynthetic process | 3.78E-03 | 0.10876 | 6 | 16 | 651 | 6320 |
| 6778 | porphyrin metabolic process | 5.34E-03 | 0.12331 | 6 | 17 | 651 | 6320 |
| 33013 | tetrapyrrole metabolic process | 5.34E-03 | 0.12331 | 6 | 17 | 651 | 6320 |
| 42168 | heme metabolic process | 5.34E-03 | 0.12331 | 6 | 17 | 651 | 6320 |
| 71577 | zinc ion transmembrane transport | 1.06E-02 | 0.17646 | 2 | 2 | 651 | 6320 |
| 10133 | proline catabolic process to glutamate | 1.06E-02 | 0.17646 | 2 | 2 | 651 | 6320 |
| 9109 | coenzyme catabolic process | 1.96E-02 | 0.27322 | 8 | 34 | 651 | 6320 |
| 6122 | mitochondrial electron transport, ubiquinol to cytochrome c | 2.04E-02 | 0.27351 | 4 | 11 | 651 | 6320 |
| 51187 | cofactor catabolic process | 2.31E-02 | 0.29914 | 8 | 35 | 651 | 6320 |
|  |  |  |  |  |  |  |  |
| *Cellular component* | |  |  |  |  |  |  |
| 5749 | mitochondrial respiratory chain complex II | 5.07E-04 | 0.005296 | 4 | 5 | 652 | 6347 |
| 45257 | succinate dehydrogenase complex (ubiquinone) | 5.07E-04 | 0.005296 | 4 | 5 | 652 | 6347 |
| 45283 | fumarate reductase complex | 5.07E-04 | 0.005296 | 4 | 5 | 652 | 6347 |
| 45281 | succinate dehydrogenase complex | 1.40E-03 | 0.013472 | 4 | 6 | 652 | 6347 |
| 45273 | respiratory chain complex II | 1.40E-03 | 0.013472 | 4 | 6 | 652 | 6347 |
| 70469 | respiratory chain | 1.34E-02 | 0.1072 | 8 | 32 | 652 | 6347 |
| 5746 | mitochondrial respiratory chain | 1.66E-02 | 0.13016 | 7 | 27 | 652 | 6347 |
|  |  |  |  |  |  |  |  |
|  |  |  |  |  |  |  |  |
| **Mitochondrial respiratory chain** | | | |  |  |  |  |
|  |  |  |  |  |  |  |  |
| *Biological process* | |  |  |  |  |  |  |
| 33108 | mitochondrial respiratory chain complex assembly | 1.25E-09 | 7.24E-07 | 16 | 28 | 651 | 6320 |
| 8535 | respiratory chain complex IV assembly | 7.27E-06 | 0.002098 | 9 | 16 | 651 | 6320 |
| 33617 | mitochondrial respiratory chain complex IV assembly | 2.08E-05 | 0.003594 | 8 | 14 | 651 | 6320 |
| 43623 | cellular protein complex assembly | 1.11E-03 | 0.062135 | 28 | 149 | 651 | 6320 |
| 34551 | mitochondrial respiratory chain complex III assembly | 1.41E-03 | 0.066947 | 4 | 6 | 651 | 6320 |
| 17062 | respiratory chain complex III assembly | 1.41E-03 | 0.066947 | 4 | 6 | 651 | 6320 |
| 17004 | cytochrome complex assembly | 1.85E-03 | 0.084343 | 5 | 10 | 651 | 6320 |
|  |  |  |  |  |  |  |  |
| *Cellular component* | |  |  |  |  |  |  |
| 32592 | integral to mitochondrial membrane | 1.20E-02 | 0.097999 | 10 | 44 | 652 | 6347 |
| 31305 | integral to mitochondrial inner membrane | 3.87E-02 | 0.27455 | 5 | 19 | 652 | 6347 |
|  |  |  |  |  |  |  |  |
|  |  |  |  |  |  |  |  |
| **Mitochondrial transport** | | |  |  |  |  |  |
|  |  |  |  |  |  |  |  |
| *Biological process* | |  |  |  |  |  |  |
| 70585 | protein localization in mitochondrion | 2.23E-03 | 0.092031 | 13 | 53 | 651 | 6320 |
| 6626 | protein targeting to mitochondrion | 2.23E-03 | 0.092031 | 13 | 53 | 651 | 6320 |
| 6839 | mitochondrial transport | 3.77E-03 | 0.10876 | 15 | 69 | 651 | 6320 |
| 65002 | intracellular protein transmembrane transport | 8.73E-03 | 0.16997 | 10 | 42 | 651 | 6320 |
| 7006 | mitochondrial membrane organization | 1.04E-02 | 0.17646 | 10 | 43 | 651 | 6320 |
| 30150 | protein import into mitochondrial matrix | 1.28E-02 | 0.20712 | 6 | 20 | 651 | 6320 |
|  |  |  |  |  |  |  |  |
| *Cellular component* | |  |  |  |  |  |  |
| 42719 | mitochondrial intermembrane space protein transporter complex | 9.21E-03 | 0.076926 | 3 | 5 | 652 | 6347 |
| 1405 | presequence translocase-associated import motor | 2.75E-02 | 0.20652 | 3 | 7 | 652 | 6347 |
| 5742 | mitochondrial outer membrane translocase complex | 4.07E-02 | 0.2837 | 3 | 8 | 652 | 6347 |
|  |  |  |  |  |  |  |  |
|  |  |  |  |  |  |  |  |
| **Cell-cell adhesion** | |  |  |  |  |  |  |
|  |  |  |  |  |  |  |  |
| *Biological process* | |  |  |  |  |  |  |
| 16337 | cell-cell adhesion | 5.13E-04 | 0.038594 | 4 | 5 | 651 | 6320 |
| 746 | conjugation | 1.10E-03 | 0.062135 | 22 | 107 | 651 | 6320 |
| 750 | pheromone-dependent signal transduction involved in conjugation with cellular fusion | 2.42E-03 | 0.09508 | 9 | 30 | 651 | 6320 |
| 19953 | sexual reproduction | 2.60E-03 | 0.095733 | 35 | 210 | 651 | 6320 |
| 749 | response to pheromone involved in conjugation with cellular fusion | 2.79E-03 | 0.10085 | 15 | 67 | 651 | 6320 |
| 7157 | heterophilic cell-cell adhesion | 4.02E-03 | 0.10876 | 3 | 4 | 651 | 6320 |
| 752 | agglutination involved in conjugation with cellular fusion | 4.02E-03 | 0.10876 | 3 | 4 | 651 | 6320 |
| 771 | agglutination involved in conjugation | 4.02E-03 | 0.10876 | 3 | 4 | 651 | 6320 |
| 7186 | G-protein coupled receptor protein signaling pathway | 4.09E-03 | 0.10897 | 10 | 38 | 651 | 6320 |
| 51704 | multi-organism process | 4.37E-03 | 0.11254 | 23 | 126 | 651 | 6320 |
| 747 | conjugation with cellular fusion | 4.65E-03 | 0.11511 | 20 | 105 | 651 | 6320 |
| 7166 | cell surface receptor linked signaling pathway | 5.00E-03 | 0.12039 | 10 | 39 | 651 | 6320 |
| 19236 | response to pheromone | 5.95E-03 | 0.13036 | 18 | 93 | 651 | 6320 |
| 48610 | reproductive cellular process | 8.50E-03 | 0.16723 | 33 | 210 | 651 | 6320 |
| 755 | cytogamy | 9.20E-03 | 0.17004 | 4 | 9 | 651 | 6320 |
| 7618 | mating | 1.06E-02 | 0.17646 | 2 | 2 | 651 | 6320 |
| 22610 | biological adhesion | 1.41E-02 | 0.21621 | 4 | 10 | 651 | 6320 |
| 7155 | cell adhesion | 1.41E-02 | 0.21621 | 4 | 10 | 651 | 6320 |
| 10033 | response to organic substance | 2.02E-02 | 0.27351 | 25 | 159 | 651 | 6320 |
|  |  |  |  |  |  |  |  |
|  |  |  |  |  |  |  |  |
| **Oxidation reduction** | | |  |  |  |  |  |
|  |  |  |  |  |  |  |  |
| *Biological process* | |  |  |  |  |  |  |
| 55072 | iron ion homeostasis | 1.90E-07 | 6.93E-05 | 19 | 50 | 651 | 6320 |
| 55066 | di-, tri-valent inorganic cation homeostasis | 2.00E-07 | 6.93E-05 | 25 | 80 | 651 | 6320 |
| 6091 | generation of precursor metabolites and energy | 1.06E-05 | 0.002612 | 44 | 221 | 651 | 6320 |
| 22900 | electron transport chain | 1.35E-05 | 0.002931 | 19 | 64 | 651 | 6320 |
| 55080 | cation homeostasis | 1.58E-05 | 0.003036 | 29 | 124 | 651 | 6320 |
| 48878 | chemical homeostasis | 3.00E-05 | 0.00473 | 31 | 141 | 651 | 6320 |
| 50801 | ion homeostasis | 3.77E-05 | 0.00544 | 30 | 136 | 651 | 6320 |
| 15891 | siderophore transport | 7.47E-05 | 0.009241 | 6 | 9 | 651 | 6320 |
| 55114 | oxidation reduction | 1.20E-04 | 0.013037 | 54 | 318 | 651 | 6320 |
| 15980 | energy derivation by oxidation of organic compounds | 2.19E-04 | 0.022342 | 30 | 149 | 651 | 6320 |
| 30005 | cellular di-, tri-valent inorganic cation homeostasis | 2.67E-04 | 0.025653 | 17 | 66 | 651 | 6320 |
| 6121 | mitochondrial electron transport, succinate to ubiquinone | 5.13E-04 | 0.038594 | 4 | 5 | 651 | 6320 |
| 6879 | cellular iron ion homeostasis | 6.86E-04 | 0.045686 | 11 | 36 | 651 | 6320 |
| 6811 | ion transport | 7.26E-04 | 0.046553 | 33 | 181 | 651 | 6320 |
| 42592 | homeostatic process | 8.71E-04 | 0.053897 | 40 | 235 | 651 | 6320 |
| 42773 | ATP synthesis coupled electron transport | 1.41E-03 | 0.066947 | 9 | 28 | 651 | 6320 |
| 42775 | mitochondrial ATP synthesis coupled electron transport | 1.41E-03 | 0.066947 | 9 | 28 | 651 | 6320 |
| 45333 | cellular respiration | 2.04E-03 | 0.088387 | 21 | 105 | 651 | 6320 |
| 51186 | cofactor metabolic process | 2.60E-03 | 0.095733 | 35 | 210 | 651 | 6320 |
| 22904 | respiratory electron transport chain | 3.10E-03 | 0.10552 | 9 | 31 | 651 | 6320 |
| 9060 | aerobic respiration | 3.22E-03 | 0.10725 | 18 | 88 | 651 | 6320 |
| 7157 | heterophilic cell-cell adhesion | 4.02E-03 | 0.10876 | 3 | 4 | 651 | 6320 |
| 752 | agglutination involved in conjugation with cellular fusion | 4.02E-03 | 0.10876 | 3 | 4 | 651 | 6320 |
| 6779 | porphyrin biosynthetic process | 3.78E-03 | 0.10876 | 6 | 16 | 651 | 6320 |
| 6783 | heme biosynthetic process | 3.78E-03 | 0.10876 | 6 | 16 | 651 | 6320 |
| 6546 | glycine catabolic process | 4.02E-03 | 0.10876 | 3 | 4 | 651 | 6320 |
| 44247 | cellular polysaccharide catabolic process | 4.02E-03 | 0.10876 | 3 | 4 | 651 | 6320 |
| 33014 | tetrapyrrole biosynthetic process | 3.78E-03 | 0.10876 | 6 | 16 | 651 | 6320 |
| 771 | agglutination involved in conjugation | 4.02E-03 | 0.10876 | 3 | 4 | 651 | 6320 |
| 16054 | organic acid catabolic process | 4.42E-03 | 0.11254 | 13 | 57 | 651 | 6320 |
| 46395 | carboxylic acid catabolic process | 4.42E-03 | 0.11254 | 13 | 57 | 651 | 6320 |
| 6778 | porphyrin metabolic process | 5.34E-03 | 0.12331 | 6 | 17 | 651 | 6320 |
| 33013 | tetrapyrrole metabolic process | 5.34E-03 | 0.12331 | 6 | 17 | 651 | 6320 |
| 42168 | heme metabolic process | 5.34E-03 | 0.12331 | 6 | 17 | 651 | 6320 |
| 15677 | copper ion import | 5.56E-03 | 0.12677 | 4 | 8 | 651 | 6320 |
| 30001 | metal ion transport | 5.75E-03 | 0.12868 | 15 | 72 | 651 | 6320 |
| 30003 | cellular cation homeostasis | 5.80E-03 | 0.12868 | 20 | 107 | 651 | 6320 |
| 41 | transition metal ion transport | 6.07E-03 | 0.13147 | 10 | 40 | 651 | 6320 |
| 9310 | amine catabolic process | 8.22E-03 | 0.1637 | 11 | 48 | 651 | 6320 |
| 272 | polysaccharide catabolic process | 9.20E-03 | 0.17004 | 4 | 9 | 651 | 6320 |
| 6873 | cellular ion homeostasis | 9.33E-03 | 0.17004 | 21 | 119 | 651 | 6320 |
| 19464 | glycine decarboxylation via glycine cleavage system | 1.06E-02 | 0.17646 | 2 | 2 | 651 | 6320 |
| 55082 | cellular chemical homeostasis | 1.02E-02 | 0.17646 | 21 | 120 | 651 | 6320 |
| 71577 | zinc ion transmembrane transport | 1.06E-02 | 0.17646 | 2 | 2 | 651 | 6320 |
| 10133 | proline catabolic process to glutamate | 1.06E-02 | 0.17646 | 2 | 2 | 651 | 6320 |
| 9063 | cellular amino acid catabolic process | 1.22E-02 | 0.19949 | 10 | 44 | 651 | 6320 |
| 35434 | copper ion transmembrane transport | 1.41E-02 | 0.21621 | 4 | 10 | 651 | 6320 |
| 6826 | iron ion transport | 1.42E-02 | 0.21648 | 5 | 15 | 651 | 6320 |
| 6825 | copper ion transport | 1.90E-02 | 0.26765 | 5 | 16 | 651 | 6320 |
| 9109 | coenzyme catabolic process | 1.96E-02 | 0.27322 | 8 | 34 | 651 | 6320 |
| 6122 | mitochondrial electron transport, ubiquinol to cytochrome c | 2.04E-02 | 0.27351 | 4 | 11 | 651 | 6320 |
| 6732 | coenzyme metabolic process | 2.03E-02 | 0.27351 | 26 | 167 | 651 | 6320 |
| 51187 | cofactor catabolic process | 2.31E-02 | 0.29914 | 8 | 35 | 651 | 6320 |
|  |  |  |  |  |  |  |  |
| *Cellular component* | |  |  |  |  |  |  |
| 5749 | mitochondrial respiratory chain complex II | 5.07E-04 | 0.005296 | 4 | 5 | 652 | 6347 |
| 45257 | succinate dehydrogenase complex (ubiquinone) | 5.07E-04 | 0.005296 | 4 | 5 | 652 | 6347 |
| 45283 | fumarate reductase complex | 5.07E-04 | 0.005296 | 4 | 5 | 652 | 6347 |
| 45281 | succinate dehydrogenase complex | 1.40E-03 | 0.013472 | 4 | 6 | 652 | 6347 |
| 45273 | respiratory chain complex II | 1.40E-03 | 0.013472 | 4 | 6 | 652 | 6347 |
| 5960 | glycine cleavage complex | 3.99E-03 | 0.035692 | 3 | 4 | 652 | 6347 |
| 70469 | respiratory chain | 1.34E-02 | 0.1072 | 8 | 32 | 652 | 6347 |
| 5746 | mitochondrial respiratory chain | 1.66E-02 | 0.13016 | 7 | 27 | 652 | 6347 |
|  |  |  |  |  |  |  |  |
|  |  |  |  |  |  |  |  |
| **Not assigned** | |  |  |  |  |  |  |
|  |  |  |  |  |  |  |  |
| *Biological process* | |  |  |  |  |  |  |
| 70887 | cellular response to chemical stimulus | 9.59E-05 | 0.011076 | 44 | 241 | 651 | 6320 |
| 42221 | response to chemical stimulus | 6.86E-04 | 0.045686 | 56 | 356 | 651 | 6320 |
| 44282 | small molecule catabolic process | 9.58E-04 | 0.057224 | 38 | 221 | 651 | 6320 |
| 55085 | transmembrane transport | 1.43E-03 | 0.066947 | 50 | 319 | 651 | 6320 |
| 9071 | serine family amino acid catabolic process | 1.41E-03 | 0.066947 | 4 | 6 | 651 | 6320 |
| 920 | cytokinetic cell separation | 2.58E-03 | 0.095733 | 6 | 15 | 651 | 6320 |
| 18065 | protein-cofactor linkage | 3.03E-03 | 0.10552 | 4 | 7 | 651 | 6320 |
| 7109 | cytokinesis, completion of separation | 3.11E-03 | 0.10552 | 5 | 11 | 651 | 6320 |
| 9249 | protein lipoylation | 4.02E-03 | 0.10876 | 3 | 4 | 651 | 6320 |
| 42044 | fluid transport | 4.02E-03 | 0.10876 | 3 | 4 | 651 | 6320 |
| 6833 | water transport | 4.02E-03 | 0.10876 | 3 | 4 | 651 | 6320 |
| 16043 | cellular component organization | 7.88E-03 | 0.15871 | 211 | 1786 | 651 | 6320 |
| 44281 | small molecule metabolic process | 1.12E-02 | 0.18513 | 123 | 991 | 651 | 6320 |
| 31670 | cellular response to nutrient | 1.37E-02 | 0.21511 | 7 | 26 | 651 | 6320 |
| 43173 | nucleotide salvage | 1.71E-02 | 0.24953 | 3 | 6 | 651 | 6320 |
| 101 | sulfur amino acid transport | 1.71E-02 | 0.24953 | 3 | 6 | 651 | 6320 |
| 42180 | cellular ketone metabolic process | 1.76E-02 | 0.25368 | 54 | 396 | 651 | 6320 |
| 6519 | cellular amino acid and derivative metabolic process | 1.83E-02 | 0.26154 | 43 | 304 | 651 | 6320 |
| 19752 | carboxylic acid metabolic process | 2.02E-02 | 0.27351 | 52 | 382 | 651 | 6320 |
| 43436 | oxoacid metabolic process | 2.02E-02 | 0.27351 | 52 | 382 | 651 | 6320 |
| 7584 | response to nutrient | 2.05E-02 | 0.27351 | 7 | 28 | 651 | 6320 |
| 6082 | organic acid metabolic process | 2.11E-02 | 0.27952 | 52 | 383 | 651 | 6320 |
| 44106 | cellular amine metabolic process | 2.18E-02 | 0.28586 | 39 | 274 | 651 | 6320 |
| 6520 | cellular amino acid metabolic process | 2.29E-02 | 0.29758 | 36 | 250 | 651 | 6320 |
|  |  |  |  |  |  |  |  |
| *Cellular component* | |  |  |  |  |  |  |
| 5743 | mitochondrial inner membrane | 5.56E-08 | 1.61E-06 | 51 | 230 | 652 | 6347 |
| 19866 | organelle inner membrane | 1.06E-07 | 2.84E-06 | 52 | 241 | 652 | 6347 |
| 5576 | extracellular region | 2.29E-07 | 5.75E-06 | 28 | 97 | 652 | 6347 |
| 5740 | mitochondrial envelope | 2.67E-07 | 6.29E-06 | 73 | 396 | 652 | 6347 |
| 31226 | intrinsic to plasma membrane | 1.62E-06 | 3.21E-05 | 14 | 33 | 652 | 6347 |
| 5887 | integral to plasma membrane | 1.84E-06 | 3.46E-05 | 12 | 25 | 652 | 6347 |
| 5886 | plasma membrane | 2.77E-06 | 4.97E-05 | 69 | 390 | 652 | 6347 |
| 31966 | mitochondrial membrane | 3.16E-06 | 5.40E-05 | 66 | 369 | 652 | 6347 |
| 31975 | envelope | 9.11E-06 | 1.43E-04 | 86 | 535 | 652 | 6347 |
| 31967 | organelle envelope | 9.11E-06 | 1.43E-04 | 86 | 535 | 652 | 6347 |
| 31970 | organelle envelope lumen | 2.22E-05 | 3.34E-04 | 16 | 50 | 652 | 6347 |
| 5758 | mitochondrial intermembrane space | 2.32E-05 | 3.35E-04 | 15 | 45 | 652 | 6347 |
| 30312 | external encapsulating structure | 1.89E-04 | 0.002372 | 24 | 108 | 652 | 6347 |
| 5618 | cell wall | 1.89E-04 | 0.002372 | 24 | 108 | 652 | 6347 |
| 9277 | fungal-type cell wall | 2.38E-04 | 0.002881 | 23 | 103 | 652 | 6347 |
| 44459 | plasma membrane part | 3.00E-04 | 0.003418 | 19 | 79 | 652 | 6347 |
| 31225 | anchored to membrane | 9.94E-04 | 0.0101 | 15 | 61 | 652 | 6347 |
| 44455 | mitochondrial membrane part | 2.76E-03 | 0.025911 | 27 | 151 | 652 | 6347 |
| 16020 | membrane | 3.50E-03 | 0.032101 | 228 | 1920 | 652 | 6347 |
| 31224 | intrinsic to membrane | 6.81E-03 | 0.059529 | 168 | 1387 | 652 | 6347 |
| 44444 | cytoplasmic part | 2.80E-02 | 0.20652 | 323 | 2915 | 652 | 6347 |
| 44425 | membrane part | 2.97E-02 | 0.21448 | 186 | 1611 | 652 | 6347 |

**Table S6: Table of yeast strains used in this study**

| **Strain** | **Genotype** | **Reference/Source** |
| --- | --- | --- |
| S288c (BY4741) | MATa; his3D1; leu2D0; ura3D0; met15D0 | Brachman et al 1998 |
| S288c (BY4742) | MATα ; his3D1; leu2D0; ura3D0 lys2D0 | Brachman et al 1998 |
| Sigma1278B | MATa | Gerry Fink |
| SK1 | MATa ura3 leu2 | Andrew Murray |
| EM93 | Mat a/α Feral strain, progenitor of S288C | Mortimer and Johnston, 1986 |
| KV34 | EM93 1A (haploid strain obtained from EM93, 1st tetrad) | Smukalla et al 2008 |
| KV35 | EM93 1B (haploid strain obtained from EM93, 1st tetrad) | Smukalla et al 2008 |
| KV36 | EM93 1C (haploid strain obtained from EM93, 1st tetrad) | Smukalla et al 2008 |
| KV37 | EM93 1D (haploid strain obtained from EM93, 1st tetrad) | Smukalla et al 2008 |
| KV2606 | KV34 containing TEF3p-FLO11 fusion | This study |
| KV2608 | KV34 containing TEF5p-FLO11 fusion | This study |
| KV2609 | KV34 containing TEF6p-FLO11 fusion | This study |
| KV2610 | KV34 containing TEF7p-FLO11 fusion | This study |
| KV2613 | KV34 containing TEF10p-FLO11 fusion | This study |
| KV2692 | KV34 FLO11-2A-YFP | This study |
| KV2693 | Sigma FLO11-2A-YFP | This study |
| KV2695 | SK1 FLO11-2A-YFP | This study |
| KV2668 | Sigma flo11::HygB | This study |
| VK2181 | KV64 tos1::LoxP-HYG-LoxP | This study |
| VK2183 | KV64 dfg16::LoxP-HYG-loxP | This study |
| VK2200 | KV64 ygr122w::LoxP-HYG-loxP | This study |
| VK2203 | KV64 rvs161::LoxP-HYG-loxP | This study |
| VK2207 | KV64 swc7::LoxP-HYG-loxP | This study |
| VK2217 | KV64 arp8::LoxP-HYG-loxP | This study |
| VK2225 | KV64 sds3::LoxP-HYG-loxP | This study |
| VK2226 | KV64 rsc2::LoxP-HYG-loxP | This study |
| VK2228 | KV64 urm1::LoxP-HYG-loxP | This study |
| VK2230 | KV64 containing TEF6p-TOS1 fusion | This study |
| VK2233 | KV64 containing TEF6p-DFG16 fusion | This study |
| VK2202 | KV2693 ygr122w::LoxP-HYG-LoxP | This study |
| VK2208 | KV2693 arp8::LoxP-HYG-LoxP | This study |
| VK2210 | KV2693 rsc2::LoxP-HYG-LoxP | This study |
| VK2211 | KV2693 urm1::LoxP-HYG-LoxP | This study |
| VK2236 | KV2693 Tos1::LoxP-HYG-LoxP | This study |
| VK2238 | KV2693 dfg16::LoxP-HYG-LoxP | This study |
| VK2242 | KV2693 sds3::LoxP-HYG-LoxP | This study |
| VK2244 | KV2693 swc7::LoxP-HYG-LoxP | This study |
| VK2252 | KV2693 rvs161::LoxP-HYG-LoxP | This study |

**S7: Table of primers used in this study**

| **Primer number** | **Use** | **Sequence** |
| --- | --- | --- |
| P67-FW | Homologuous sequence to amplify G418 cre-lox (pUG6) | CAGGTCGACAACCCTTAAT |
| P68-RV | Homologuous sequence to amplify G418 cre-lox (pUG6) | GTGGATCTGATATCACCTA |
| P69-RV | Check insert KANMX cassette | GCACGTCAAGACTGTCAAGG |
| 2711-flo11-f | To amplify locus from deletion collection | TTCAAGGCATCATCGCAATA |
| 2712-flo11-r | To amplify locus from deletion collection | TGGGATGATTTTTGGTGGTT |
| 2867-FLO11-int | Primer to check ORF replacement by KANMX | GAAAACCCAAAGCCGAGTTA |
| 2932-FLO11-int | Primer to check ORF replacement by KANMX | TTGAACCCAAGAAACGGAAG |
| 2933-Flo11-f3 | Primer to check ORF replacement by KANMX | AATTGGGATTCAAGGCATCA |
| 2934-FLO11-int-rev3 | Primer to check ORF replacement by KANMX | TTGGGACAGCCATTAACGAT |
| 2964-flo11-replace-hyg-F | Replace locus with HYGB cassette | ATGCAAAGACCATTTCTACTCGCTTATTTGGTCCTTTCGCTTCTATTTAACTCGGCTTTG-TGCCATCTTTGTACAGCTTGCCT |
| 2965-flo11-replace-hyg-R | Replace locus with HYGB cassette | TTAGAATACAACTGGAAGAGCGAGTAGCAACCACATAAAGTTTCCAAGAACCTTGATATTA-CGCAGAGCCGTGGCAGG |
| 2966-flo11-hyg-check-F | Primer to check ORF replacement by HYG B | GATAACTCATAGACTTACCAGTACAAATTG |
| 2967-flo11-hyg-check-F | Primer to check ORF replacement by HYG B | AATGTCATAGAGTTACCAATTGGGATTCAAG |
| 2994-FLO11-out-F | Primer to check ORF replacement by HYGB | TCCGTTCTCTTCTGATGAGGT |
| 2995-FLO11-out-R | Primer to check ORF replacement by HYGB | TGTTACGTCTCTTTCCGTCT |
| 2996-SFL1-replace-F | Forward primer to delete SFL1 gene | CAGAGGGTCCTAAGACAGCACAAATCAGTTATATAGAAAAAAAGAAGAAAAAATCATGCAGGTCGACAACCCTTAAT |
| 2997-SFL1-replace-R | Reverse primer to delete SFL1 gene | ACATACAGTGAGGTGCTTTGAACTTTTAGACAACTAGAGATTAAAAAGGCAAAGAGTGGATCTGATATCACCTA |
| 2998-SFL1-check-F | Primer to check ORF replacement by KANMX | CACATACGCATACGCACACA |
| 2999-SFL1-check-R | Primer to check ORF replacement by KANMX | TCACAAGGATCAGGAGGAAAA |
| 3000-FLO11pm-del-HYG-F | Primer to check ORF replacement by HYGB | TTTGCGGCAATACAAATCTATAGCCGGGATCACCGACCTTGTTATATTCTTCGGAGACAT-TGCCATCTTTGTACAGCTTGCCT |
| 3001-FLO11pm-del-HYG-R | Primer to check ORF replacement by HYGB | GACGAGGGATAATAACTGATGAATAGGGTGCTTTTTATACGTTTATTTTAGGAAGCAAGA-CGCAGAGCCGTGGCAGG |
| 3003-FLO11-check-R | Primer to check ORF replacement by HYGB | TGCGTATATGGATTTTTGAGG |
| 3043-FLO11-FMDV2A-yEVenus-F | Primer to construct cleavable YFP-Flo11p | GCTAATATCAAGGTTCTTGGAAACTTTATGTGGTTGCTACTCGCTCTTCCAGTTGTATTC-GGC AGT GGA GTT AAG CAG ACC TTG AAT TTC GAT CTG CTA AAG TTA GCT GGT GAC GTC GAG TCT AAT CCT GGC CCA -GGTGACGGTGCTGGTTTA |
| 3044-FLO11-TaV2A-yEVenus-F | Primer to construct cleavable YFP-Flo11p | GCTAATATCAAGGTTCTTGGAAACTTTATGTGGTTGCTACTCGCTCTTCCAGTTGTATTC-GGC AGT GGA GAG GGC AGA GGA AGT CTG CTA ACA TGC GGT GAC GTC GAG GAG AAT CCT GGC CCA-GGTGACGGTGCTGGTTTA |
| 3081-HYG-SigmaFLO11-F | Primers to mark Sigma FLO11 locus with HYG | AATACATATTCATTTACATATTTTTTAGTTCAAAAATTATCTTTTACCTTTTTTTACTTT-TGCCATCTTTGTACAGCTTGCCT |
| 3082-HYG-SigmaFLO11-R | Primers to mark Sigma FLO11 locus with HYG | ACTTGTTCTTGAATAAGCTATCAGATGGTCAAAATAGGGCTAGAAGAAAAATGGTAAAAT-CGCAGAGCCGTGGCAGG |
| 3083-SigFLO11-cassette-F | F primer to amplify SigmaFLO11-HYG cassette (use with R primer 3082) | CACACTATGCAAAGACCATTTC |
| 3084-SigmaFLO11check-R | Primer to anneal specifically to Sigma FLO11 (use with F primer 2994). | TTTTAGGTCAATGTTTTCTTC |
| 3085-S288cFLO11check-R | Primer to anneal specifically to S288c FLO11 (use with F primer 2994). | CTTCAGGTCAATATTTTCTTT |
| P36 | RT-PCR ACT1 Forward | CTCCACCACTGCTGAAAGAGAA |
| P37 | RT-PCR ACT1 Reverse | CCAAGGCGACGTAACATAGTTTT |
| P35 | RT-PCR FLO11 Reverse 2 | CTTGCATATTGAGCGGCACTAC |
| P51 | RT-PCR FLO11 Forward 3 | CACTTTTGAAGTTTATGCCACACAAG |

| 4399-TEF_OE-TOS1-Fw | Primer to introduce TEF6 promoter | CCATAAAATATCTAAAACCTAACGGAATTTGAACGAAAAACAGCTGAAGCTTCGTACGC |
| --- | --- | --- |
| 4400-TEF_OE-TOS1-Rv | Primer to introduce TEF6 promoter | CTGAAGAAAACAAACCGACTAATGCGGTCATGGAAAGCTTTTGTAACATTTTTCTAGAAAACTTAG |
| 4401-TOS1_ck-Fw | Primer to check promoter replacement | ATCTCTCGAACTTTGCTAGTTAAAC |
| 4402-TEF_OE-DFG16-Fw | Primer to introduce TEF6 promoter | CACTCACAAGCATTTTTTGTTGCGGAACGTCTTTACGTGCAAGATTACAGCTGAAGCTTCGTACGC |
| 4403-TEF_OE-DFG16-Rv | Primer to introduce TEF6 promoter | CTAAAGTGAGGAGGTAGTAAAAATGCAATCTTATGATCAT TTTTCTAGAAAACTTAG |
| 4404-DFG16_ck-Fw | Primer to check promoter replacement | GTGAAGGTAACACAATCGATGTA |
| 4407-DFG16DEL-Fw | Primer to replace locus with HYGB | CGATGTAATTGATATCAGATTTACTAGCCCCGTTGGATTGCAGCTGAAGCTTCGTACGC |
| 4408-DFG16DEL-Rv | Primer to replace locus with HYGB | GACCGGTTGTTATGCGATCTGACGTAAAGTGGAACTCATTGCATAGGCCACTAGTGGATCTG |
| 4480-DFG16-ck-Rev | Primer to check deletion | GTCGTAATGCGGTGGTCTTTC |
| 4431-YGR122wDel-Fw | Primer to replace locus with HYGB | CTCAATTACGGGCTGTGGTAACACTTGAGATCTTGTCTTCCCCAGCTGAAGCTTCGTACGC |
| 4432-YGR122wDel-Rv | Primer to replace locus with HYGB | CACCAGATCGGTAGACTATAATTGATTCTCACTCCAACACACGCATAGGCCACTAGTGGATCTG |
| 4433-YGR122wDEL_ck-Fw | Primer to check deletion | GTGCGCTCGAATAGTGCCG |
| 4434-RVS161DEL-Fw | Primer to replace locus with HYGB | CAGGCAGACGTGAGAGAAGAAAAGGAAACGCTGTGATATAGCAGCTGAAGCTTCGTACGC |
| 4435-RVS161DEL-Rv | Primer to replace locus with HYGB | CCCGTTTGGCTGATTACGGATCACGTTCAAATTGGTAAATCGCATAGGCCACTAGTGGATCTG |
| 4436-RVS161DEL_ck-Fw | Primer to check deletion | GCGGAGAAGATGTTGCCACG |
| 4451-SDS3DEL-Fw | Primer to replace locus with HYGB | GCTTATGTATTGATCATTTCGCTTGAGATTTCAGGAAGCAGCTGAAGCTTCGTACGC |
| 4452-SDS3DEL-Rv | Primer to replace locus with HYGB | CATCTAATTATGATGTATATTCTAGCCCTTCTTTAGTACAACGCATAGGCCACTAGTGGATCTG |
| 4453-SDS3DEL_ck-Fw | Primer to check deletion | GAGATTGGGTACGTTTGGATG |
| 4457-SWC7DEL-Fw | Primer to replace locus with HYGB | GCTTTGTTTCTGCATTTACAGTTAATTCTCTACTTCGTTAGCAGCTGAAGCTTCGTACGC |
| 4458-SWC7DEL-Rv | Primer to replace locus with HYGB | GCTGTAATGATATCACCTTGTTCAAAGACAAAGACTAATTGGGCATAGGCCACTAGTGGATCTG |
| 4459-SWC7DEL_ck-Fw | Primer to check deletion | GCAAAGCGTATGCGTAGTCT |
| 4460-ARP8DEL-Fw | Primer to replace locus with HYGB | CTAGTCAATAGTACATAAATACAGGGATACAATCGCACGCAGCTGAAGCTTCGTACGC |
| 4461-ARP8DEL-Rv | Primer to replace locus with HYGB | GTTGTTGCACCATGCATGTTACCATCATAGCCGTCTAAGCGCATAGGCCACTAGTGGATCTG |
| 4462-ARP8DEL_ck-Fw | Primer to check deletion | GGCCGTGCAAGATGACTTAT |
| 4463-RSC2DEL-Fw | Primer to replace locus with HYGB | CACTGTAATAAGCACATTTGTAGGTGCTCTTGCACTTGGCAGCTGAAGCTTCGTACGC |
| 4464-RSC2DEL-Rv | Primer to replace locus with HYGB | CGGATAGTTTATTTCACTTTAGTTGTAGCAACTTACTGGGCATAGGCCACTAGTGGATCTG |
| 4465-RSC2DEL_ck-Fw | Primer to check deletion | CGTTCAAAAGACAGTATGTTC |
| 4466-URM1DEL-Fw | Primer to replace locus with HYGB | CAATACTGATTTCTGATACTAAAACGAGATAGGTTAATAGCCAGCTGAAGCTTCGTACGC |
| 4467-URM1DEL-Rv | Primer to replace locus with HYGB | CTATTACTTTTTATACTATCTGCATATTACCTACGTTATCGCATAGGCCACTAGTGGATCTG |
| 4468-URM1DEL_ck-Fw | Primer to check deletion | CTGGCAGACGGTATGGAGAG |
| 4482-TOS1Del-Fw | Primer to replace locus with HYGB | CATAAAATATCTAAAACCTAACGGAATTTGAACGAAAAAACAGCTGAAGCTTCGTACGC |
| 4483-TOS1Del-Rv | Primer to replace locus with HYGB | AAAAAAAGGAAAATTTTATTGGTTAATAAGAAACGATCGAGCATAGGCCACTAGTGGATCTG |
| 4479-TOS1-ck-Rev | Primer to check deletion | CAGCTTGGGTACAAGCACAAG |
| 147-Hyg-Con-R1 | Primer to check deletion (to use for all constructs, reverse primer, anneals in HYGB) | TCGACAGACGTCGCGGTGAGTT |
|  |  |  |

**Supplemental File S1: Extent of over- and underexpression of differentially expressed genes in *flo11* mutant versus WT in liquid and solid medium (excel file).**
